# An Elastocaloric Polymer with Ultra‐High Solid‐State Cooling via Defect Engineering

**DOI:** 10.1002/advs.202518106

**Published:** 2025-12-12

**Authors:** Zhaohan Yu, Duo Xu, Zumrat Usmanova, Chuwei Ye, Aditya Swarnkar, Emma Scott, Sizhe Huang, Siyuan Rao, Xinyue Liu, Svetlana V. Boriskina, Ruobing Bai, Shaoting Lin

**Affiliations:** ^1^ Department of Mechanical Engineering Michigan State University East Lansing MI 48824 USA; ^2^ Department of Mechanical Engineering Massachusetts Institute of Technology Cambridge MA 02139 USA; ^3^ Department of Mechanical and Industrial Engineering Northeastern University Boston MA 02115 USA; ^4^ Department of Biomedical Engineering Binghamton University Binghamton NY 13902 USA; ^5^ Department of Chemical Engineering and Materials Science Michigan State University East Lansing MI 48824 USA

**Keywords:** elastocaloric polymers, solid‐state cooling, strain‐induced crystallization, tetra‐arm poly(ethylene glycol), topological defects

## Abstract

Elastocaloric polymers, whose performance typically relies on phase transformation between amorphous chains and crystalline domains, offer a promising alternative to traditional refrigeration technologies. While engineering polymer‐network architecture has shown the potential to boost elastocaloric performance, the role of topological defects remains unexplored despite their prevalence in real polymers. This study reports a defect‐engineering approach in end‐linked star polymers (ELSPs) that enables an adiabatic temperature change of up to 8.14 ± 1.76 °C at an ambient temperature above 65 °C, showing an enhancement of 39% compared to ELSPs with negligible defects. This defect‐regulated solid‐state cooling is attributed to two competing effects of dangling‐chain defects on strain‐induced crystallization (SIC) and temperature‐induced crystallization (TIC), synergistically regulating the adiabatic temperature change. Specifically, increasing dangling‐chain defects monotonically lowers ELSPs’ mechanical performance at high temperatures due to suppressed SIC, but nonmonotonically impacts the mechanical performance at low temperatures due to the competition between suppressed SIC and enhanced TIC.

## Graphical abstract

This work reports a defect‐engineering strategy that enables a record‐high adiabatic temperature change of up to 8.14 ± 1.76 °C in elastomers at an elevated temperature above 65 °C. Combined experimental and theoretical efforts reveal that increasing dangling‐chain defects nonmonotonically impacts the elastocaloric cooling performance of polymers due to the competition between suppressed strain‐induced crystallization and enhanced temperature‐induced crystallization.

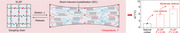

## 1 Introduction

Strain‐induced crystallization (SIC) is a ubiquitous phenomenon commonly observed in polymeric materials such as elastomers^[^ ^1^, ^2^, ^3^, ^4^, ^5^, ^6^, ^7^ ^]^ and gels.^[^ ^8^, ^9^, ^10^, ^11^, ^12^ ^]^ When these polymers are subjected to uniaxial mechanical strain, their amorphous polymer chains transform into highly oriented and aligned crystalline domains through either intermolecular or intramolecular interactions. Generally, SIC is classified into two types based on their reversibility. A poly(vinyl alcohol) hydrogel (PVA), widely used in diverse engineering applications, particularly in the biomedical field, is one representative example exhibiting irreversible SIC.^[^ ^10^ ^]^ When subjected to mechanical strain, the PVA chains with abundant hydroxyl groups form oriented and aligned crystalline domains. These crystalline domains typically cannot be reverted to the original amorphous chains,^[^ ^13^ ^]^ manifested by the large stress–strain hysteresis of the hydrogel.^[^ ^10^ ^]^ Leveraging this irreversibility, researchers have developed mechanical training methods to engineer aligned nanofibrillar architecture in synthetic PVA hydrogels, successfully achieving synergistic muscle‐like properties.^[^ ^10^, ^14^, ^15^, ^16^, ^17^ ^]^ In contrast to PVA, a natural rubber undergoes reversible SIC,^[^ ^3^, ^4^, ^5^, ^6^, ^7^ ^]^ enabling rapid reinforcement under mechanical load and ≈100% recovery within seconds when unloaded. Beyond natural rubber, the recently emerging slide‐ring polyethylene glycol (PEG) also exhibits reversible SIC in its gel state.^[^ ^8^, ^9^, ^11^ ^]^ Unlike traditional toughening mechanisms such as Mullins effect^[^ ^18^, ^19^, ^20^, ^21^, ^22^ ^]^ and viscoelasticity^[^ ^23^, ^24^, ^25^ ^]^ that involve permanent damage or require substantial recovery time, toughening by reversible SIC does not rely on large mechanical hysteresis. The anisotropic crystalline domains around the crack tip promote crack blunting, facilitate crack deflection, and thus resist crack growth.^[^ ^1^, ^8^, ^26^, ^27^, ^28^ ^]^ These features of SIC provide an attractive approach to enhance the fracture‐ and fatigue‐resistance of soft materials.

In addition to its attractive role in enhancing mechanical properties, SIC further uniquely enables an emerging cooling technology known as *solid‐state elastocaloric cooling*.^[^ ^29^, ^30^, ^31^ ^]^ When a polymeric material is rapidly stretched, parts of its amorphous chains transform into ordered crystalline domains, releasing heat and raising the material's temperature. Over time, heat dissipates into the surrounding environment, and the material's temperature gradually returns to its original state. Once the material is further instantaneously unstretched, its temperature decreases, and the material absorbs heat from the ambient environment, achieving a cooling effect. Different from traditional cooling by refrigeration, elastocaloric cooling does not involve toxic chemicals and offers potential for zero‐greenhouse gas emission, representing a promising alternative with superior environmental friendliness and inherent solid‐state characteristics.^[^ ^30^, ^31^, ^32^, ^33^ ^]^

Despite this promising potential of solid‐state elastocaloric cooling, so far, its commercialization has been hindered by the low cooling efficiency of existing materials due to their insufficient SIC. As the most common elastocaloric polymer, natural rubber achieves only ≈15% SIC when stretched to six times its initial length at room temperature, leading to an adiabatic temperature change of 3.5 °C at a temperature ≈55 °C.^[^ ^1^ ^]^ This low SIC and elastocaloric performance is likely due to the uncontrollable, many *topological defects* in the polymer network. These topological defects, such as cyclic loops and dangling chains, are inherently present in nearly all polymers.^[^ ^34^, ^35^, ^36^ ^]^ They are usually induced by finite reaction efficiency (e.g., 95% in typical commercial polymers) due to impurities and suboptimal synthesis conditions, or the intrinsic network connectivity of a polymer, even with 100% reaction efficiency. Recent studies have shown that rational engineering of topological defects can greatly improve the elastocaloric performance. Zhang et al. demonstrated the dependence of elastocaloric cooling performance on chain‐length uniformity in commercial triblock poly(styrene‐b‐ethylene‐co‐butylene‐b‐styrene) thermoplastic elastomers.^[^ ^31^ ^]^ Hartquist et al. reported that an elastomer of end‐linked star polymer with negligible topological defect achieves up to 50% SIC at temperatures over 55 °C, significantly boosting the elastocaloric effect.^[^ ^1^ ^]^ Despite these advances, the role of topological defects in regulating a polymer's elastocaloric behavior has remained largely unexplored. In particular, new opportunities have yet to be explored due to the close relationship between elastocaloric cooling and the mechanical properties of a polymer network, as the latter is not always negatively impacted by topological defects. Whereas cyclic‐loop and dangling‐chain defects generally reduce the elastic modulus of a polymer network,^[^ ^37^ ^]^ they provide either weakening or toughening in the crack resistance, depending on the specific type and density.^[^ ^38^, ^39^, ^40^, ^41^ ^]^ This duality arises from the competition between the weakening due to inactive chains and the toughening due to increased effective chain lengths.^[^ ^39^ ^]^ Therefore, to fundamentally improve the elastocaloric cooling performance, it is urgent to understand the quantitative role of topological defects and synergize the associated mechanisms via a defect‐engineering approach.

Here, we report a defect‐engineering approach in end‐linked star polymers (ELSPs) that enables an adiabatic temperature change of up to 8.14 ± 1.76 °C at an elevated temperature above 65 °C, representing a 39% enhancement compared to ELSPs with negligible defects and a 350% enhancement compared to natural rubber. We utilize tetra‐arm polyethylene glycol (PEG) systems as our model ELSPs. We investigate strain‐ and temperature‐induced crystallization in ELSPs with various controlled dangling‐chain defects, focusing on their effects on mechanical and elastocaloric properties. We conduct thermo‐mechanical characterization and develop a thermodynamic model to systematically analyze the impact of dangling‐chain defects on crystallization under different stretch ratios and temperatures. Our experimental and theoretical analyses reveal two competing effects: suppression of SIC hinders the amorphous chain alignment and crystalline domain orientation, and promotion of temperature‐induced crystallization (TIC) enhances the polymer network flexibility. We further perform mechanical tests to characterize the large deformation, damage, fracture, and fatigue of ELSPs with controlled dangling‐chain defects. At temperatures above the ELSPs’ melting point, defects decrease the stress level, increase the hysteresis ratio, decrease the fracture toughness, and increase the fatigue threshold. At temperatures below the melting point, defects decrease the stress level and increase the fatigue threshold but show nonmonotonic effects on the hysteresis ratio and fracture toughness. Finally, elastocaloric experiments on ELSPs with controlled defects show an intriguing defect‐dependent adiabatic temperature change Δ*T* resulting from the interplay between suppressed SIC and enhanced TIC. These results highlight the critical role of topological defects in regulating the cooling performance of elastocaloric polymers. This work is hoped to advance the fundamental understanding of topological defects in elastocaloric polymers, as well as to pave the way for developing next‐generation efficient, flexible, and solid‐state cooling materials.

## 2 Results

### 2.1 ELSPs with Controlled Dangling‐Chain Defects

**Figure 1:**
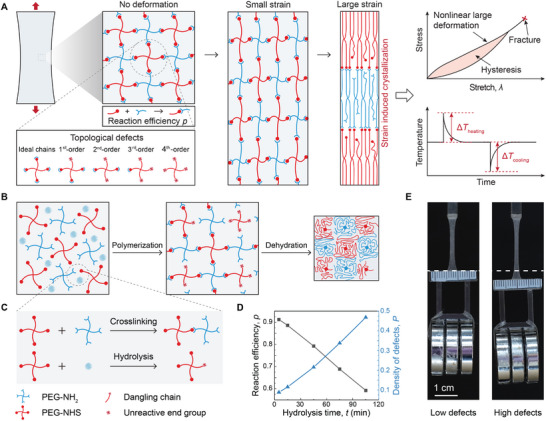
End‐linked star polymers (ELSPs) with dangling‐chain defects. A) Schematic illustration of ELSPs with controlled dangling‐chain defects under various strain conditions: no deformation, small strain, and large strain, where SIC occurs. SIC will, in turn, influence the mechanical behavior of ELSPs, including nonlinear large deformation, hysteresis, and fracture, as well as their elastocaloric performance, characterized by adiabatic temperature changes during rapid stretching and releasing. B) Schematic illustration of ELSPs synthesis with controlled dangling‐chain defect density. Tetra‐arm PEG‐NH_2_ reacts with tetra‐arm PEG‐NHS to form an end‐linked star hydrogel with various orders of dangling‐chain defects. After a dehydration process, the hydrogel transforms into an elastomer. C) The NHS ester in tetra‐arm PEG‐NHS can either react with NH_2_ easter to form cross‐links or hydrolyze in the presence of water, the latter resulting in unreactive end groups and ultimately creating dangling‐chain defects in the material system. By controlling the hydrolysis time, ELSPs with different densities of defects can be obtained. D) Calibrated reaction efficiency *p* and density of defects *P* as a function of hydrolysis time *t*. E) Elongation of ELSPs with low and high defect density under a constant load (60 g). Scale bar in (E) is 1 cm.

We prepare ELSPs with controlled densities of dangling‐chain defects by finely tuning the reaction efficiency based on an A‐B type tetra‐arm PEG system (**Figure** **1**A). The PEG hydrogel consists of tetra‐amine‐terminated PEG macromers (PEG‐NH_2_) and tetra‐NHS‐terminated PEG macromers (PEG‐NHS), cross‐linked via amine‐NHS bonds. Both macromers have a molecular weight of 20000 g mol^−1^, with each arm weighing 5000 g mol^−1^. Different from conventional randomly cross‐linked polymer networks, the A‐B type tetra‐PEG network, pioneered by Sakai et al., exhibits unique characteristics such as monodisperse chain length and negligible molecular entanglements.^[^ ^42^, ^43^, ^44^, ^45^ ^]^ As illustrated in Figure 1A, when subjected to deformation, the tetra‐PEG chains are initially coiled and gradually uncoiled at small strains, while at larger strains they align along the stretching direction, which facilitates SIC. The synthesis of the PEG hydrogel follows the reported protocol^[^ ^46^ ^]^ (Figure 1B). The hydrogel then undergoes an evaporation process to obtain solvent‐free ELSPs with a crystalline melting temperature *T*_m_ of ≈45 °C.^[^ ^1^, ^47^ ^]^ During the preparation process, the NHS group simultaneously reacts with the amine group and undergoes hydrolysis in an aqueous solution. The former interlinks the PEG macromers, but the latter inhibits the NHS‐amine reaction (Figure 1C). By controlling the hydrolysis time, we can regulate the reaction efficiency *p*. This introduces controlled densities and types of dangling‐chain defects in the ELSPs system.

The number of dangling chains on one macromer defines the order of dangling‐chain defects (Figure 1A). The relationship between the density of defects and hydrolysis time is calibrated by a defect‐network elastic model,^[^ ^46^ ^]^ which predicts the shear modulus of a polymer network with various defects. Using this model, the reaction efficiency *p* can be inferred from the measured shear modulus and calibrated following μ(*p*)/μ(*p* = 1) = 1 − (5/3)(1 − *P*)^3^ *P* − 7(1 − *P*)^2^ *P* ^2^ − (17/3)(1 − *P*)*P* ^3^ − *P* ^4^, where *P* = *pP* ^3^ + 1 − *p* indicates the probability of a dangling end for one arm of the macromer, representing the density of defects.^[^ ^39^, ^46^ ^]^ Here, *μ*(*p*) is the measured shear modulus of the polymer network with reaction efficiency *p* (Figure S1, Supporting Information), and μ(*p* = 1) = *NkT* denotes the shear modulus of an ideal polymer network without defect predicted by the phantom network model,^[^ ^48^ ^]^ where *N* is the number of elastically effective chains per unit volume. This equation also denotes the fundamental difference between the cross‐linking density (*N*) and the defect density (*P*): *N* is governed by the polymer chain length *n* through individual macromers, but *P* is governed by the order and density of dangling‐chain defects through the reaction efficiency *p* and hydrolysis time *t* ^[^ ^39^, ^46^, ^49^ ^]^ (see detailed discussions in Supporting Information). As the hydrolysis time increases from 5 to 105 min, the reaction efficiency *p* decreases from 0.91 to 0.59, and the density of defects *P* increases from 0.09 to 0.47 (Figure 1D), leading to reduced modulus and strength. Under the same load, the elongation of an ELSP increases with the defect density (Figure 1E). The density of dangling‐chain defects in ELSPs was experimentally quantified using a fluorescamine assay (Figure S2, Supporting Information), validating the density of defects estimated by the defect‐network elastic model.

### 2.2 Impacts of Dangling‐Chain Defects on SIC and TIC

Crystallization in ELSPs primarily occurs in two forms: SIC and TIC. SIC takes place when the polymer undergoes tensile strain, aligning amorphous chains into oriented crystalline domains.^[^ ^47^ ^]^ In contrast, TIC takes place during a cooling process, where amorphous chains randomly coil and rearrange, forming non‐oriented crystalline structures.^[^ ^50^, ^51^ ^]^ Here we conduct structural characterization including differential scanning calorimetry (DSC) and in situ X‐ray scattering under various stretching and temperature conditions to quantify the density (crystallinity) *χ* and the size d of crystalline domains, as well as the start‐to‐start spacing *L* between crystalline domains. In addition, we adopted a mean‐field, continuum model (Figure S3, Supporting Information) to describe the defect‐regulated SIC and the corresponding uniaxial stress–strain response at different temperatures. The model builds on the pioneering work by Flory on SIC in natural rubbers^[^ ^52^ ^]^ and adapts the recent continuum theory of Rastak and Linder^[^ ^53^ ^]^ (see details in Supporting Information). Previous studies have demonstrated the applicability of this approach in describing defect‐regulated elasticity in polymer networks with defect densities as high as over 50%.^[^ ^37^, ^39^ ^]^ This model quantitatively predicts the crystallinity in ELSPs under different stretch ratios and temperatures, enabling us to decouple the defect‐regulated SIC and TIC processes.

**Figure 2:**
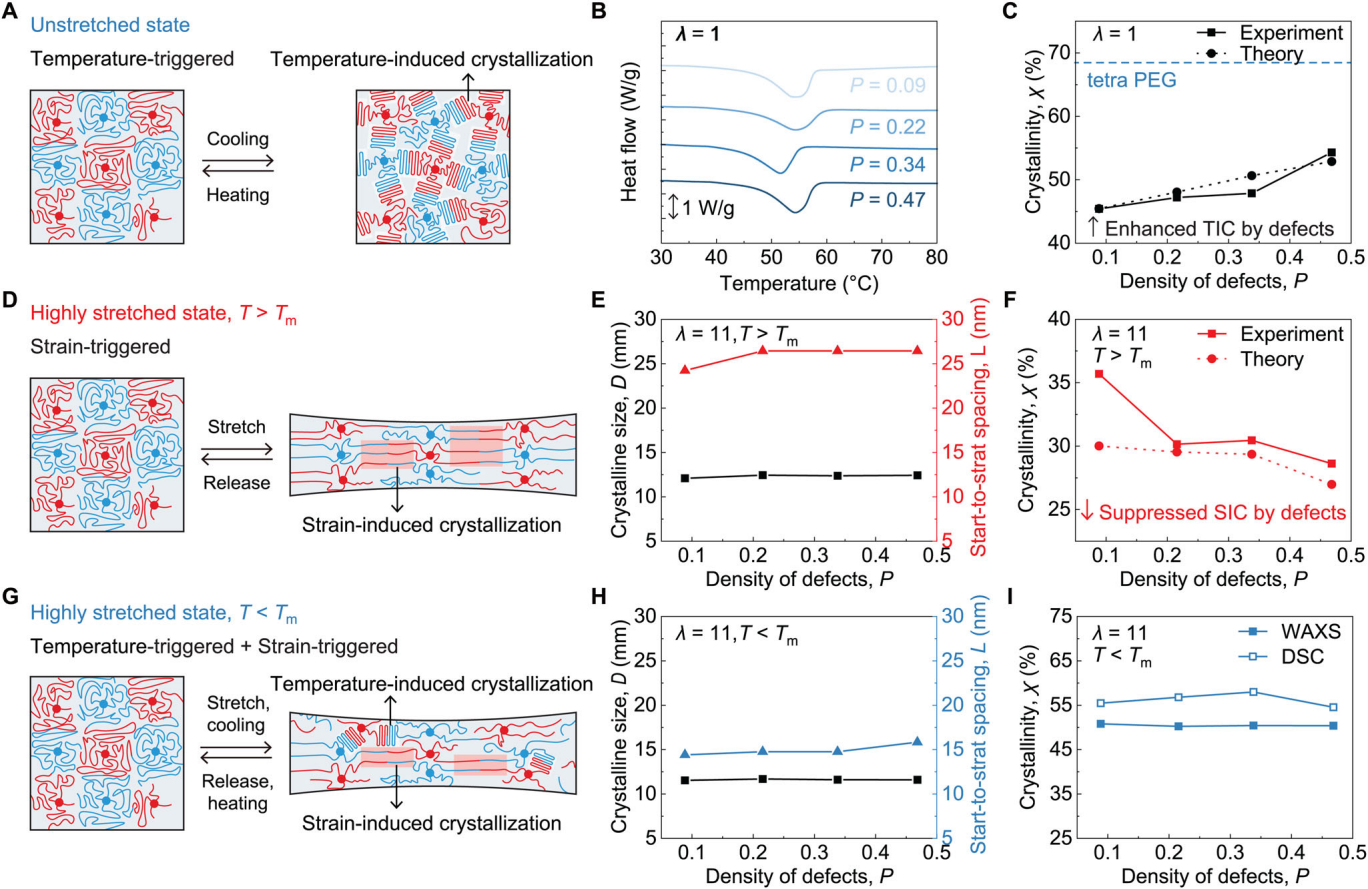
Temperature‐induced crystallization (TIC) and strain‐induced crystallization (SIC) in ELSPs with various dangling‐chain defects. A) Schematic illustration of TIC formation in the unstretched state. B) DSC curves of ELSPs with various density of defects *P* at the unstretched state (*λ* = 1). C) Crystallinity *χ* of ELSPs with various density of defects *P* at unstretched state (*λ* = 1). The black solid line denotes the experimental results, while the black dashed line denotes the theoretical calculation. The blue dashed line shows the crystallinity of pristine uncrosslinked tetra PEG. D) Schematic illustration of SIC formation in ELSPs in a highly stretched state at a temperature above *T*_m_. E) Crystalline domain size *D* and start‐to‐start spacing *L* of ELSPs with various density of defects *P* subjected to large stretch (*λ* = 11) at *T* = 60 °C above *T*_m_. F) Crystallinity *χ* of ELSPs with various density of defects *P* subjected to large stretch (*λ* = 11) at *T* = 60 °C above *T*_m_. The solid line denotes the experimental results, while the dashed line denotes the theoretical calculation. G) Schematic illustration of the crystallization in ELSPs in a highly stretched state at a temperature below *T*_m_. H) Crystalline domain size *D* and start‐to‐start spacing *L* of ELSPs with various density of defects *P* subjected to large stretch (*λ* = 11) at *T* = 40 °C below *T*_m_. I) Crystallinity *χ* of ELSPs with various density of defects *P* subjected to large stretch (*λ* = 11) at *T* = 40 °C below *T*_m_.

We first examine the crystallization of unstretched ELSPs at temperatures below the melting point (*T* < *T*_m_), where the crystalline domains are primarily governed by TIC (**Figure** **2**A). Our DSC results, shown in Figure 2B,C, reveal that the crystallinity of unstretched ELSPs (*λ* = 1) increases from 45.4% to 54.3% as the density of dangling‐chain defects increases from 0.09 to 0.47, agreeing well with our theoretical prediction. Additionally, we measure the crystallinity of uncross‐linked tetra‐arm PEG to be 68%, significantly higher than that of ELSPs with various dangling‐chain defects. This indicates that the elasticity of cross‐linked polymer networks in ELSPs suppresses TIC, which depends on the flexibility of polymer chains.^[^ ^54^, ^55^ ^]^

We then investigate the crystallization of highly stretched ELSPs at temperatures above the melting point (*T* > *T*_m_), where the crystalline domains are predominantly governed by SIC (Figure 2D). Different from the crystallization characterization at *T* < *T*_m_, we adopt in situ X‐ray scattering measurements, because DSC cannot maintain the pre‐applied stretch during heating and therefore cannot capture the crystallinity of ELSPs in the stretched state. This approach allows us to quantify the crystalline size 𝐷 and start‐to‐start spacing *L* of ELSPs under controlled stretch ratios.^[^ ^56^ ^]^ As shown in Figure 2E, as the density of dangling‐chain defects increases from 0.09 to 0.47, the crystalline size 𝐷 remains relatively stable at ≈12.40 nm (Figure S4, Supporting Information), while the start‐to‐start spacing *L* slightly increases from 24.23 to 26.44 nm (Figure S5, Supporting Information). The peaks in the wide‐angle X‐ray scattering (WAXS) intensity profile represent the amorphous and crystalline contributions. By fitting the peaks using Gaussian or pseudo‐Voigt functions,^[^ ^57^, ^58^ ^]^ we quantify the SIC of ELSPs with controlled dangling‐chain defects (Figure S6, Supporting Information). As shown in Figure 2F, when the density of dangling‐chain defects increases from 0.09 to 0.47, the SIC in highly stretched ELSPs (*λ* = 11) decreases from 35.7% to 28.6%, agreeing with our theoretical prediction. The suppression of SIC by dangling‐chain defects is likely due to their interference with the alignment of amorphous chains and the orientation of crystalline domains, both essential for effective SIC.^[^ ^1^ ^]^

We further study the crystallization of highly stretched ELSPs at temperatures below the melting point (*T* < *T*_m_), where both TIC and SIC coexist (Figure 2G). Similar to highly stretched ELSPs at *T* > *T*_m_, the crystalline size d remains relatively stable at ≈11.60 nm (Figure 2H; Figure S4, Supporting Information) but is slightly smaller than that at *T* > *T*_m_ (Figure 2E). This is possibly due to crystalline contraction during cooling, as demonstrated by recent crystalline characterizations of tetra‐arm PEGs with negligible defects.^[^ ^47^ ^]^ As the density of dangling‐chain defects increases, the start‐to‐start spacing *L* slightly increases from 14.41 to 15.83 nm (Figure 2H; Figure S5, Supporting Information) but remains much lower than that at *T* > *T*_m_ (Figure 2E; Figure S5, Supporting Information). The slight reduction in the crystalline size d and the notable decrease in the start‐to‐start spacing *L* indicate a substantial increase in the overall crystallinity *χ*, as confirmed by measurements from both WAXS (Figure 2I; Figures S4 and S8, Supporting Information) and DSC (Figure 2I; Figure S9, Supporting Information). Intriguingly, different from the monotonic dependence of SIC on defect density at *T* > *T*_m_ (Figure 2F), the overall crystallinity *χ* in highly stretched ELSPs (*λ* = 11) at *T* < *T*_m_ remains nearly constant as the defect density increases (Figure 2I). This behavior reflects an interplay between the suppression of SIC and the promotion of TIC induced by defects.

### 2.3 Impacts of Dangling‐Chain Defects on Mechanical Properties

Next, we perform mechanical tests to characterize the stress–strain response, hysteretic dissipation, fracture, and fatigue of ELSPs with controlled dangling‐chain defects, specifically focusing on the effects of dangling‐chain defects above *T*_m_ (*T* = 60 °C) and below *T*_m_ (*T* = 25 °C).

**Figure 3:**
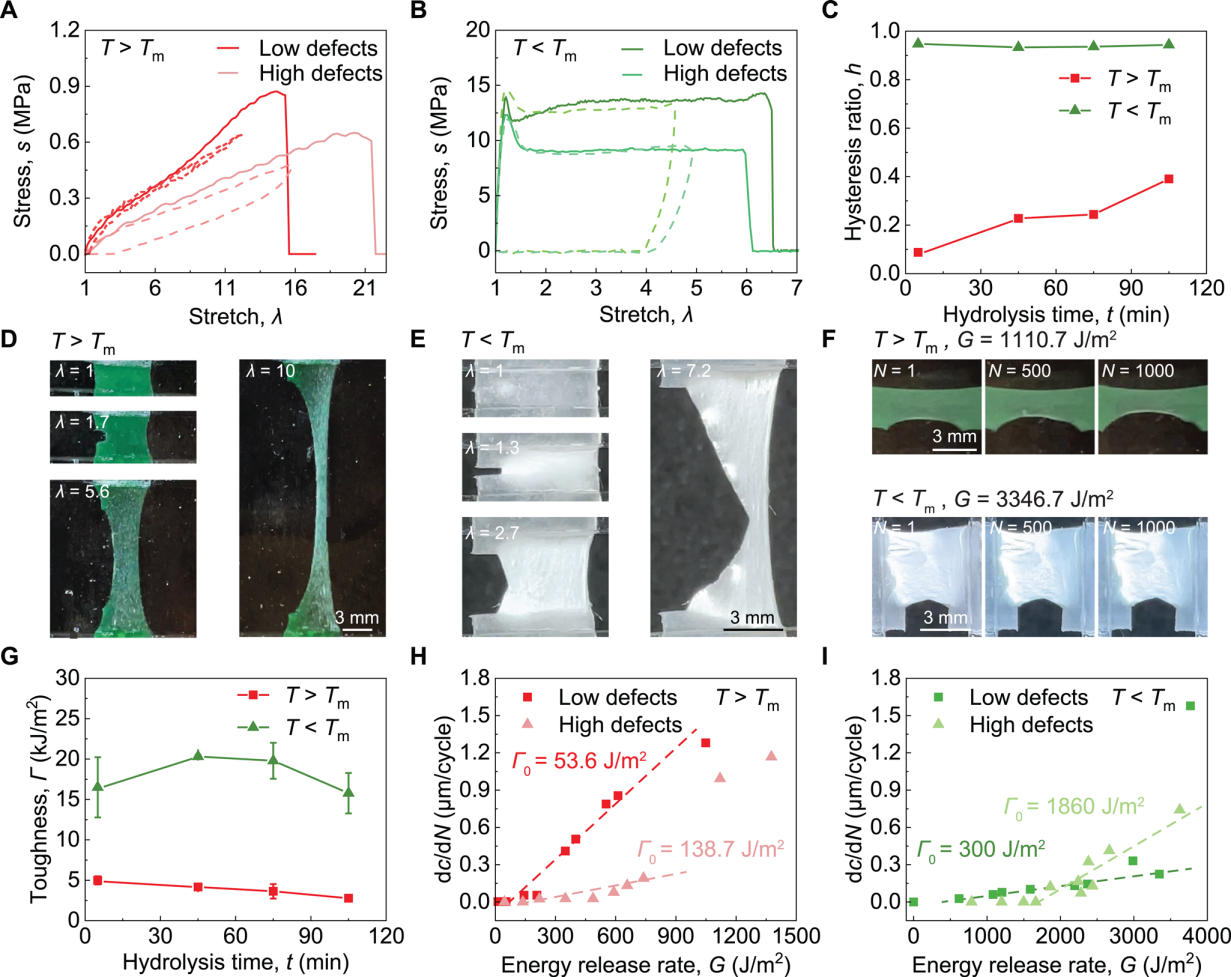
Mechanical properties of ELSPs at temperatures below and above *T*_m_. A) Nominal stress *s* versus stretch *λ* curves of ELSPs with high (*P* = 0.47) and low (*P* = 0.09) defect density at *T* = 60 °C (*T* > *T*_m_). The solid line denotes monotonic loading, while the dashed line denotes cyclic loading. B) Nominal stress versus stretch curves of ELSPs with high (*P* = 0.47) and low (*P* = 0.09) defect density at *T* = 25 °C (*T* < *T*_m_). The solid line denotes monotonic loading, while the dashed line denotes cyclic loading. C) Hysteresis ratio *h* of ELSPs with varying hydrolysis time *t* at *T* = 60 °C (*T* > *T*_m_) and *T* = 25 °C (*T* < *T*_m_). D) Images of crack propagation in ELSPs with low defect density (*P* = 0.09) subjected to stretch ratios of 1, 1.7, 5.6, and 10 at *T* = 60 °C (*T* > *T*_m_). E) Images of crack propagation in ELSPs with low defect density (*P* = 0.09) subjected to stretch ratios of 1, 1.3, 2.7, and 7.2 at *T* = 25 °C (*T* < *T*_m_). F) Images of fatigue crack propagation in ELSPs with low defect density (*P* = 0.09) subjected to applied energy release rate of *G* = 1110.7 J m^−2^ at *T* = 60 °C above *T*_m_ and applied energy release rate of *G* = 3346.7 J m^−2^ at *T* = 25 °C below *T*_m_. G) Fracture toughness *Г* of ELSPs with various hydrolysis time *t* at *T* = 60 °C (*T* > *T*_m_) and *T* = 25 °C (*T* < *T*_m_). The error bar variability may stem from minor residual stress introduced during the drying process. H) Fatigue crack extension per cycle d*c*/d*N* as a function of applied energy release rate *G* of ELSPs with high (*P* = 0.47) and low (*P* = 0.09) defect density at *T* = 60 °C (*T* > *T*_m_). The fatigue thresholds *Г*_0_ of ELSPs with high (*P* = 0.47) and low (*P* = 0.09) defect density are identified as 138.7 and 53.6 J m^−2^, respectively. I) Fatigue crack extension per cycle d*c*/d*N* as a function of the applied energy release rate *G* of ELSPs with high (*P* = 0.47) and low (*P* = 0.09) defect density at *T* = 25 °C (*T* < *T*_m_). The fatigue thresholds *Г*_0_ of ELSPs with high (*P* = 0.47) and low (*P* = 0.09) defect density are identified as 1860 and 300 J m^−2^, respectively. Scale bars in (D), (E), and (F) are 3 mm. Error bars in (G) denote standard deviations.

At high temperature (60 °C), unstretched ELSPs are in the rubbery phase, where the crystallization is mainly governed by SIC, modulating their elasticity, hysteresis, fracture, and fatigue. We measure the monotonic nominal stress‐stretch (*s*‐*λ*) curves of ELSPs with high (*P* = 0.47) and low (*P* = 0.09) defect densities at *T* = 60 °C. As shown in **Figure** **3**A, the increase of dangling‐chain defect density decreases the Young's modulus from 188.7 to 64.0 kPa and increases the stretchability from 1471.2% to 2021.6%. The former is attributed to the reduced density of elastically active chains^[^ ^46^ ^]^ and the latter to the increased effective chain length.^[^ ^39^ ^]^ Our experiments also reveal a reduction in strength from 0.87 to 0.65 MPa with increasing defects, possibly due to the suppression of SIC by defects via their interference with the alignment of amorphous chains and the orientation of crystalline domains.

The impact of defects on the stress–strain hysteresis ratio *h* is quantified by the cyclic stress‐stretch curves of ELSPs with high (*P* = 0.47) and low (*P* = 0.09) defect density at *T* = 60 °C. As shown in Figure 3C, the presence of defects significantly increases *h* from 0.09 to 0.40, suggesting increased network damage and dissipation. Indeed, dangling‐chain defects promote irreversible energy dissipation by the Mullins effect^[^ ^18^, ^19^, ^20^, ^21^, ^22^ ^]^ and suppress reversible dissipation by SIC.^[^ ^21^ ^]^

Crack resistance of ELSPs under monotonic loading at *T* = 60 °C is characterized by the measured fracture toughness. Figure 3D visualizes the initiation, blunting, and propagation of a sharp crack in the ELSPs with a low defect density (*P* = 0.09). As *P* increases from 0.09 to 0.47, the fracture toughness decreases from 4.98 to 2.78 kJ m^−2^ (Figure 3G). Although the stress–strain hysteresis generally enhances the fracture toughness,^[^ ^20^ ^]^ the results here indicate that the reduction in reversible dissipation from SIC plays a more dominant role in lowering the toughness. Additionally, crack resistance under cyclic loading at *T* = 60 °C is characterized by fatigue tests (Figure 3F; Figure S10, Supporting Information). In contrast to the results of fracture toughness, the fatigue threshold *Γ*_0_ of the high‐defect ELSP (*P* = 0.47) reaches 138.7 J m^−2^, more than two times higher than that of the low‐defect ELSP (*P* = 0.09) of 53.6 J m^−2^. This increase is attributed to the increased effective chain length, a key parameter for enhancing fatigue resistance.^[^ ^59^, ^60^ ^]^

At low temperature (25 °C), unstretched ELSPs are already in the semicrystalline phase. Further crystallization under stretching is governed by both SIC and TIC, synergistically modulating the elastic‐plastic responses, hysteresis, fracture, and fatigue of ELSPs. This is indicated by the same mechanical tests on ELSPs with high (*P* = 0.47) and low (*P* = 0.09) defect densities at *T* = 25 °C. In monotonic stress‐stretch curves (Figure 3B), increasing the defect density (from *P* = 0.09 to 0.47) decreases the Young's modulus (from 103.8 to 83.7 MPa), yielding strength (from 13.65 to 9.15 MPa), and strength (from 14.28 to 12.31 MPa). These trends are similar to those at *T* > *T*_m_, due to the reduced density of elastically active chains.^[^ ^46^ ^]^ By contrast, unlike *T* > *T*_m_, increasing the defect density slightly reduces the stretchability of ELSPs (from 635.6% to 592.0%) at *T* = 25 °C. Furthermore, at *T* = 25 °C, both high‐ and low‐defect samples exhibit large hysteresis ratios (*h* > 0.90) with negligible difference (Figure 3C), possibly due to the dominant effect of irreversible damage under stretch in the semicrystalline polymer. At *T* = 25 °C, a crack is much sharper and more rapidly propagating compared to *T* > *T*_m_ (Figure 3E). The fracture toughness shows a nonmonotonic trend with the increasing defect density (Figure 3G), first increasing from 16.50 to 20.16 kJ m^−2^, reaching the maximum at *P* = 0.22, and then decreasing to 15.78 kJ m^−2^. This nonmonotonic trend is attributed to the competing effects of defect‐suppressed SIC and defect‐promoted TIC. As shown in Figure 3I and Figure S11 (Supporting Information), the fatigue threshold *Γ*_0_ of the high‐defect ELSP (*P* = 0.47) is 1860 J m^−2^, significantly higher than that of the low‐defect ELSP (*P* = 0.09, *Γ*_0_ = 300 J m^−2^). This trend is consistent with the observation above *T*_m_, possibly due to the increased effective chain length in ELSPs with high defect density.

### 2.4 Impacts of Dangling‐Chain Defects on Elastocaloric Performance

**Figure 4:**
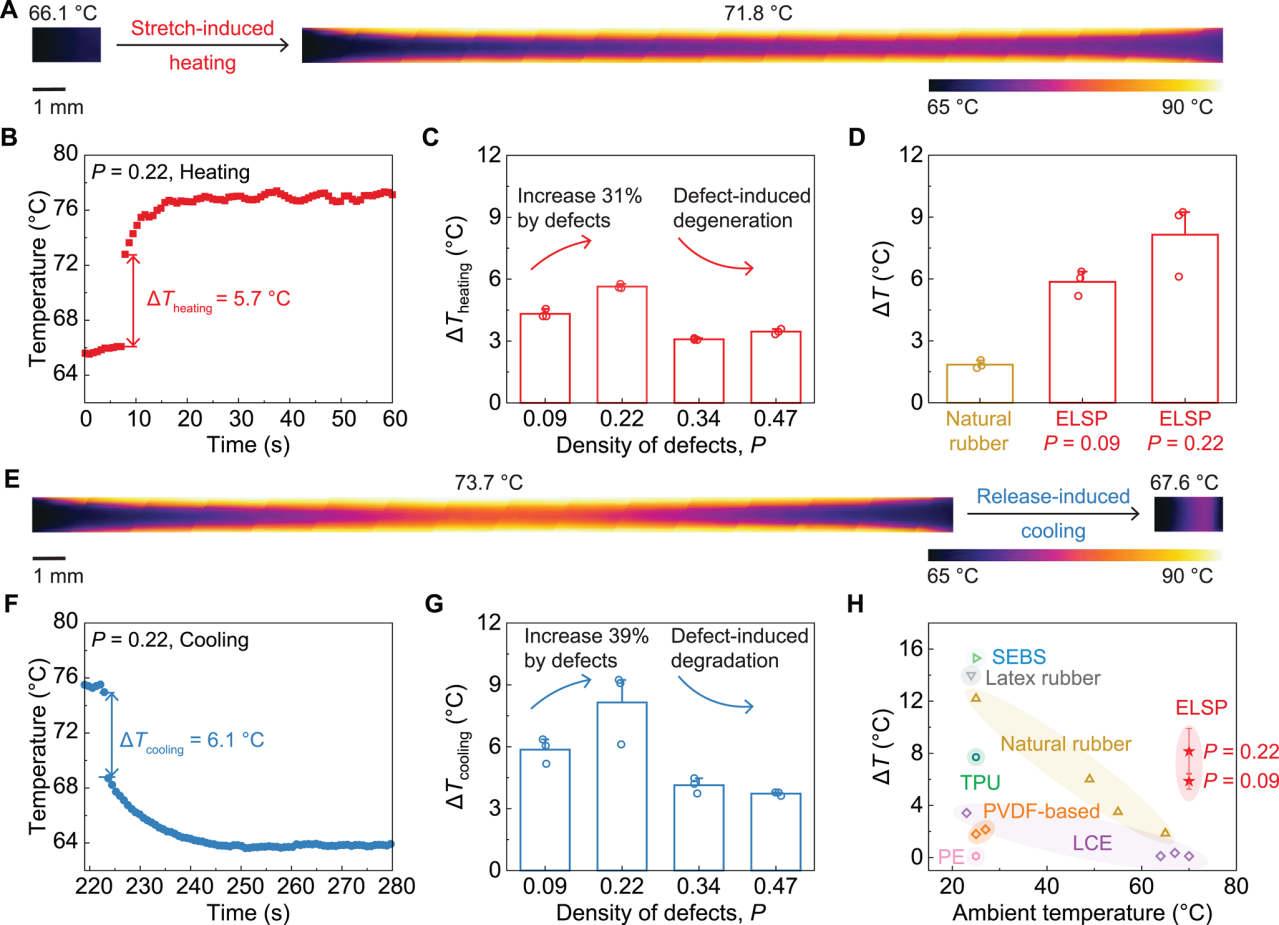
Elastocaloric effect of ELSPs with various dangling‐chain defects. A) Thermal images of ELSP with moderate defect density (*P* = 0.22) during the adiabatic stretching process. B) Measured surface temperature of the ELSP with moderate defect density (*P* = 0.22) during instantaneous stretching. Δ*T* denotes the adiabatic temperature change that occurs during adiabatic stretching or releasing. C) Adiabatic temperature change Δ*T_heating_* of ELSPs with various density of defects *P* from 0.09 to 0.47 during instantaneous stretching. D) Adiabatic temperature change of natural rubber and ELSP with various density of defects during adiabatic release at ambient temperatures above 65 °C. E) Thermal images of ELSP with moderate defect density (*P* = 0.22) during the adiabatic releasing process. F) Measured surface temperature of the ELSP with moderate defect density (*P* = 0.22) during instantaneous releasing. G) Adiabatic temperature change Δ*T_cooling_* of ELSPs with various density of defects *P* from 0.09 to 0.47 during instantaneous releasing. H) Comparison of elastocaloric performance at different ambient temperatures among various elastocaloric polymers under tensile testing. Scale bars in (A) and (E) are 1 mm. Error bars in (C) and (G) denote standard deviations.

Elastocaloric materials have been widely studied (**Figure** **4**D; Tables S2 and S3, Supporting Information), with natural rubber often serving as a model system, which exhibits limited SIC (≈20%) and poor fatigue properties.^[^ ^61^, ^62^ ^]^ Our recent work has highlighted an ELSP with ultrahigh SIC of up to 50% and an adiabatic temperature change Δ*T* of ≈9.3 °C, surpassing natural rubber (3.5 °C) by ≈3.5 times.^[^ ^1^ ^]^ However, this experimental value remains much lower than the theoretical prediction given by $\Delta T=\frac{\rho \Delta H}{c}\Delta \chi -\frac{1}{c}T\Delta S_{chain}$, with *ρ* being the density, *c* the volumetric specific heat, Δ*H* the latent heat of crystalline domains, *T* the temperature in Kelvin, and Δ*S_chain_* the configurational entropy by stretching polymer chains.^[^ ^1^, ^30^, ^63^ ^]^ This gap cannot be simply explained by existing thermodynamic models that typically neglect the impact of topological defects, which are ubiquitous in real material systems. Therefore, there is a crucial need to study the impact of topological defects on SIC to modulate the elastocaloric effects in polymers. Unlike cross‐linking density (Table S1, Supporting Information), whose effects are relatively well understood (a higher cross‐linking density reduces stretchability, suppresses nonlinear elasticity, and hinders elastocaloric performance), the role of topological defects remains largely unexplored (see details in Supporting Information). In the following, we perform experiments to systematically investigate their impact on the elastocaloric performance of ELSPs.

As shown in Figure 4A and Figure S12 (Supporting Information), an unstretched ELSP is placed in an environmental chamber equilibrated to ≈65 °C. The sample is then rapidly stretched to a stretch ratio of 11 at 400 mm s^−1^ and held at the deformed state for up to 180 s. Surface temperature measurements are taken using an infrared thermal camera for both undeformed and deformed states. To ensure accuracy, surface temperatures are recorded at five distinct locations on the sample (Figure S13, Supporting Information), and their average value is used as the representative surface temperature. As shown in Figure 4B, upon stretching, the average surface temperature increases rapidly from 66.1 to 71.8 °C within one second, followed by a slower increase over 10 s. This rapid temperature increase upon stretching is attributed to the short nucleation time for SIC (≈20 ms^[^ ^64^ ^]^). Therefore, we take the short‐term temperature rise as the measured adiabatic heating temperature Δ*T_heating_* during instantaneous stretch. Similarly, we take the short‐term temperature decrease as the measured adiabatic cooling temperature Δ*T_cooling_* during instantaneous release.

Using this experimental setup, we measure the adiabatic heating temperature Δ*T_heating_* in ELSPs with controlled dangling‐chain defects. Summarized in Figure 4C and Figure S14 (Supporting Information), as the defect density *P* increases from 0.09 to 0.47, Δ*T_heating_* exhibits a nonmonotonic trend, reaching a peak value at a moderate density (*P* = 0.22). The adiabatic cooling temperature Δ*T_cooling_* is similarly measured. When a highly stretched, moderate‐defect ELSP (*P* = 0.22) is instantaneously released (Figure 4E), its average surface temperature decreases from 73.7 to 67.6 °C (Figure 4F), giving an adiabatic cooling temperature Δ*T_cooling_* of 6.1 °C. Similar to Δ*T_heating_* , Δ*T_cooling_* follows a nonmonotonic trend peaking at *P* = 0.22 (Figure 4G; Figure S14, Supporting Information), achieving an increase of adiabatic temperature change of 39% compared to ELSPs with negligible defects (*P* = 0.09). The nonmonotonic dependency of Δ*T_heating_* and Δ*T_cooling_* on defect density suggests that dangling‐chain defects do not necessarily degenerate the elastocaloric cooling performance of the polymer. Beyond dangling‐chain defects, we anticipate that cyclic‐loop defects, if engineered rationally, may yield a more pronounced enhancement, since cyclic loops do not introduce inactive polymer chains that suppress the elasticity.^[^ ^39^ ^]^ Notably, our defect‐engineered ELSP achieves an adiabatic temperature change of up to 8.14 ± 1.76 °C at an ambient temperature above 65 °C, outperforming all existing elastocaloric polymers (Figure 4H; Table S3, Supporting Information), including natural rubber (1.8 °C at an ambient temperature above 65 °C) (Figure 4D; Figure S15, Supporting Information). Furthermore, our experiments show that low‐defect ELSPs exhibit stable elastocaloric performance for over 100 cycles (Figure S16, Supporting Information), indicating excellent long‐term durability. These findings suggest the promising potential of defect‐engineered ELSPs for both high‐temperature and long‐term elastocaloric applications, which will be explored in our future studies.

## 3 Discussion

In this study, we systematically investigate the impact of topological defects on crystallization in end‐linked star polymers (ELSPs) and how these effects manifest in the mechanical properties and elastocaloric performance. Through a combination of experimental characterization and theoretical analysis, we reveal that topological defects induce two competing effects: the suppression of SIC by impeding the simultaneous alignment of amorphous chains and the orientation of crystalline domains, and the promotion of TIC by enhancing the flexibility of the polymer network. These competing effects, governed by integrated strain and temperature modulations, collectively dictate an ELSP's mechanical properties and elastocaloric performance. Mechanical characterizations of ELSPs with controlled dangling‐chain defects show that, above the melting point, defects reduce the stress level, increase the hysteresis ratio, lower the fracture toughness, and enhance the fatigue threshold. Below the melting point, similar trends occur in the stress level and fatigue threshold, but the hysteresis ratio and the fracture toughness exhibit nonmonotonic dependencies. This behavior reflects the defect‐mediated balance between chain mobility and chain orientation, where enhanced chain mobility promotes TIC and hindered alignment suppresses SIC. Correspondingly, elastocaloric experiments reveal an intriguing defect‐dependent adiabatic temperature change Δ*T* that mirrors this competition.

These findings provide fundamental insights into the complex role of topological defects in polymer systems. It is hoped that the comprehensive understanding of these interactions will not only advance the fundamental knowledge of topological defects in elastocaloric polymers but also inform the design of next‐generation efficient flexible solid‐state cooling materials through defect engineering. Additionally, the superior performance of defect‐engineered ELSPs at elevated temperatures makes them ideal candidates for high‐temperature elastocaloric applications, including waste‐heat management and localized cooling in high‐power electronic and industrial equipment, as well as wearable cooling devices for individuals exposed to extreme heat. Beyond these, their reversible amorphous‐crystalline transitions also open opportunities for thermally driven actuation and hybrid energy‐harvesting systems, where part of the thermal energy can be converted into mechanical work.

Beyond practical applications, further theoretical extensions could deepen the molecular‐level understanding of defect‐regulated crystallization. The present model assumes homogeneous distributions of cross‐links and defects, while its free‐energy formulation can also be extended to full‐field boundary‐value problems with prescribed stochastic distributions of inhomogeneous cross‐links or defects. In addition, computational tools such as molecular dynamics (MD) and Monte Carlo (MC) simulations^[^ ^65^, ^66^ ^]^ could be incorporated to resolve the detailed molecular processes at the length scale of polymer chains. These simulations will also shed light on alternative approaches to link the chain‐level and the macroscopic deformation beyond the Arruda‐Boyce eight‐chain model,^[^ ^67^ ^]^ such as the micro‐sphere model.^[^ ^68^, ^69^ ^]^ Moreover, the kinetics of the polymer melt‐crystal phase transformation, which can be described by the well‐established Allen‐Cahn equation,^[^ ^70^ ^]^ needs to be investigated in future studies to help unveil any rate dependencies in defect‐regulated SIC, stress–strain, and elastocaloric behaviors.

Future work will further extend this framework by combining advanced in situ characterization with multiscale modeling and molecular dynamics simulations to provide more direct molecular‐level insights into defect‐regulated crystallization dynamics. In parallel, the impact of topological defects on thermal conductivity and the nonequilibrium elastocaloric performance of ELSPs under cyclic loading will be explored to further expand their potential applications.

## Conflict of Interest

The authors declare no conflict of interest.

## Author Contributions

Z.Y., D.X., and Z.U. contributed equally to this work. S.L. conceived the idea. S.L., R.B., and S.V.B. supervised the research. Z.Y. and A.S. prepared the ELSPs samples. Z.Y. performed mechanical characterizations and fluorescamine assay, and carried out DSC characterizations. D.X., and S.H. performed the in situ X‐ray scattering measurements. D.X., E.S., C.Y., and Z.Y. carried out elastocaloric cooling experiments. Z.U. and R.B. carried out theoretical calculations. Z.Y., D.X., Z.U., C.Y., A.S., E.S., S.H., S.R., S.V.B., R.B., X. Liu, and S.L. analyzed and interpreted the results. Z.Y. drafted the manuscript with input from all other authors. S.L. reviewed the manuscript.

## Supporting information



Supporting Information

## Data Availability

The data that support the findings of this study are available from the corresponding author upon reasonable request.
