# Supplementary material for: An Elastocaloric Polymer with Ultra‐High Solid‐State Cooling via Defect Engineering
Source: Adv Sci (Weinh). 2025 Dec 12;13(10):e18106. doi: 10.1002/advs.202518106 (PMC12915172; doi:10.1002/advs.202518106)
Supplement: Supplementary file 1 — Supporting Information [file ADVS-13-e18106-s001.pdf]

Supplementary Materials for

An Elastocaloric Polymer with Ultra-High Solid-State Cooling via Defect Engineering

The PDF file includes:

Supplementary Notes

Figs. S1 to S16

Tables S1 to S3

References

Supplementary Methods

Materials

Unless otherwise indicated, all chemical reagents and solvents were purchased and used without further purification. Tetra-amine-terminated PEG (4arm-PEG-NH_2, Laysan Bio) and tetra-NHS-terminated PEG (4arm-PEG-NHS, Laysan Bio) were purchased from Laysan Bio. Phosphate buffered saline (P4417, tablet, Sigma-Aldrich), phosphate-citrate buffer (P4809, tablet, Sigma-Aldrich), sodium hydroxide (NaOH, 0583, pellets, VWR international), and hydrochloric acid (HCl, 258148, Sigma-Aldrich) were obtained from Sigma-Aldrich. Food colors were purchased from Spice Supreme. Molds for the hydrogel were made from acrylic sheets (8560K171, McMaster-Carr), which were cut into the desired shape and size using a laser cutter (Fusion Maker 12, Epilog). The PH was measured using a benchtop pH meter (Fisherbrand FE150).

ELSPs synthesis

We synthesized the ELSPs with controlled densities of dangling-chain defects by dehydrating end-linked star hydrogel following the reported protocols (*1-3*). The synthesis began by reacting tetra-amine-terminated PEG (PEG-NH_2) with tetra-NHS-terminated PEG (PEG-NHS), both with molecular weights of 20000 g/mol. First, a phosphate buffer solution with an ionic strength of 100 mM was prepared by dissolving one tablet of phosphate buffered saline in 300 mL deionized water, and the pH was adjusted to 7.4 with sodium hydroxide (NaOH) (*4*). A phosphate-citric buffer solution with an ionic strength of 100 mM was prepared by dissolving one tablet of phosphate-citric buffer in 150 mL deionized water, and the pH was adjusted to 5.8 with hydrochloric acid (HCl). Next, 100 mg of PEG-NH_2 was dissolved and mixed thoroughly in 1 mL of phosphate buffer solution. Similarly, 100 mg of PEG-NHS was dissolved and mixed thoroughly in 1 mL of phosphate-citric buffer solution. To tune the reaction efficiency p, the hydrolysis time t of the PEG-NHS solution was controlled, during which the NHS groups became inactive by hydrolyzing the activated esters. Subsequently, the two solutions were thoroughly mixed, giving a final concentration of 50 mg/mL for each macromer. The resulting mixture was then poured into a 30 mm × 15 mm × 1.5 mm acrylic mold and placed in a closed chamber for 12 hours to form amide bonds. The reaction efficiency was evaluated in the gel state by comparing the as-prepared shear modulus to the defect-network elastic theory (*1*). After the hydrogel was formed, it was placed on a silicone oil-lubricated acrylic plate to prevent adhesion between the sample and the plate. It was then dehydrated in a fume hood at room temperature for 36 hours. Finally, the obtained ELSPs were carefully heated above the melting temperature T_m (around 45 °C) (*2*) and cooled to room temperature to alleviate any residual stress.

Fluorescamine assay

The density of dangling-chain defects in ELSPs was experimentally quantified using a fluorescamine assay. In this system, the defects originate from partial hydrolysis of NHS groups before crosslinking between PEG-NH_2 and PEG-NHS. Hydrolysis of NHS groups renders them unreactive toward amines, leaving residual NH_2 groups in the polymer network. The concentration of these unreacted amines serves as a quantitative indicator of the density of defects.

Fluorescamine, a non-fluorescent reagent that reacts selectively with primary amine groups to produce fluorescent fluorophores (**Fig. S2A**), was used to determine the residual NH_2 content (*5, 6*). Calibration solutions were prepared by dissolving PEG-NH_2 (20 kDa) in PBS buffer (PH = 8.5) at various known amine concentrations. The relative fluorescence units (RFU) were recorded using a Synergy H1 plate reader with excitation/emission wavelengths of 390 nm / 475 nm (**Fig. S2C**). A strong linear correlation between RFU and amine amount was established below 4 nmol (**Fig. S2D**).

Using this calibration, ELSP samples prepared with different hydrolysis times were tested (**Fig. S2B**) to quantify their residual amine contents (**Fig. S2E, F**). Longer hydrolysis times resulted in greater NHS hydrolysis and higher levels of unreacted NH_2 groups, eventually reaching a plateau. The normalized defect density was obtained by dividing the residual amine content by the plateau value (**Fig. S2G**). The defect densities determined by this method were consistent with those predicted by the defect-network elastic model (**Fig. S2H**), confirming the reliability of this quantitative approach.

<u>DSC measurement</u>

Differential Scanning Calorimetry (DSC) was conducted on the ELSPs with various defects and stretches to determine the melting temperature T_m and heat flow characteristics, which are indicative of crystallinity. A differential scanning calorimeter (DSC 2000, TA Instruments) was used to measure heat flow as a function of time and temperature. Approximately 5 mg of ELSPs were placed in aluminum hermetic pans for the measurements. The temperature was cycled between 25 °C and 100 °C at a rate of 5 °C/min during both the heating and cooling process. The melting temperature was derived from the endothermic peak observed during heating. The crystallinity of ELSPs was calculated by $\chi = (\Delta H_M/\Delta H_M^0) \times 100\%$, where ΔH_M represents the enthalpy of fusion for the sample obtained from the DSC curve, and ΔH_M^0 is the enthalpy of fusion for 100% crystalline polymer, which is 197 J/g (*7*).

<u>X-ray scattering</u>

Small-angle X-ray scattering (SAXS) and wide-angle X-ray scattering (WAXS) were performed with a large area 2D Dectris Pilatus3R 300K detector (X-ray Diffraction Shared Experimental Facility at Massachusetts Institute of Technology). During the measurements, the vacuum chamber was pumped to 0.08 mbar to minimize fluctuations in background intensity. The alignment of the sample and beamstop was carefully checked before taking measurements. For the X-ray test of stretched ELSPs, the samples were prepared by stretching to a specific stretch in a 55 °C oil bath, as detailed in the mechanical test section. To fix the sample in the stretched state and prevent any recovery during the test, the samples were secured in a custom-designed acrylic mold using Krazy glue. Their positions were adjusted to ensure that the stretching direction was aligned vertically with the sample holder. The samples remained in direct contact with the heating apparatus and were conductively heated by a Linkam HFSX350 temperature stage (Scientific Instruments). SAXS and WAXS tests for each sample were conducted at 30 °C, 40 °C, 50 °C, and 60 °C, with sufficient time (300 seconds) allowed before each test to ensure the samples reached a steady state.

To obtain the crystallinity of ELSPs, the raw 2D SAXS and WAXS patterns were converted to 1D intensity profiles by average over all azimuthal angles. While some reported crystallinity measurements average over a narrow azimuthal range, we counter that this method leads to inflated crystallinity values in anisotropic samples. To mitigate this error, we chose to average over all azimuthal angles, a method validated by a 2D convolution scheme designed to differentiate between orientation in amorphous and crystalline domains. The average intensity measured during scattering was plotted against the scattering angle 2θ, and its relationship to the scattering vector q is $q = 4\pi\, sin(2\theta/2)/\Lambda$, where Λ represents the x-ray wavelength, which is 1.54 Å. This value relates to the characteristic interplanar d-spacing via Bragg's law, which is defined as $2d \sin\theta = n\Lambda$, where d refers to the interplanar spacing and n refers to the diffraction order.

The effective crystalline sizes D can be measured through WAXS. By identifying the half width of the maximum diffraction peak β, D can be calculated following Scherrer's equation as $D = k\Lambda/\beta \cos\theta$, where k represents a dimensionless shape factor and is set as 0.94, and θ is the Bragg angle.

The average distance between adjacent crystalline domains L can be measured through SAXS. It's estimated from the critical vector corresponding to the peak intensity q_{max}, following the Bragg expression as $L = 2\pi/q_{max}$.

The crystallinity was evaluated by fitting Gaussian or Pseudo-Voigt curves to the crystalline and amorphous peaks identified from the 1D intensity profile after subtracting the background scattering intensity. The peak fitting operation was performed using a curve fitting and peak fitting software (Fityk 1.3.1). By summing the areas under the fitted curves, the crystallinity χ can be calculated as $\chi = A_C/(A_C + A_A)$, where A_C refers to the area under the crystalline peak and A_A refers to the area under the amorphous peaks (**Fig. S6**) (*8*).

<u>Mechanical testing</u>

All the mechanical tests were performed using a U-Stretch device (Univert, CellScale) in horizontal mode. For tests below the melting temperature, they were performed in ambient air at room temperature with a 100 N load cell. Tests conducted above the melting temperature were performed in a silicone oil bath at 55 °C, using a 10 N load cell. The loading speed was fixed at 60 mm/min for all mechanical tests. During the test, the U-stretch device measured force F and displacement ΔH. Nominal stress s was calculated as $s = F/(WT)$, where W represents the width and T is the thickness of the sample in the undeformed state. The stretch λ was evaluated as $\lambda = (H + \Delta H)/H$, with H being the initial height of the undeformed sample. Samples were secured using grooved jaw clamps under mechanical pressure.

Measurement of stress-stretch curves and hysteresis ratio. We first conducted tensile tests under a monotonic loading to measure the stress-stretch curves of ELSPs up to failure. Separately, to compare the hysteresis behavior, a single cycle of loading and unloading test was performed on a set of ELSPs with controlled maximum stretch λ_a. The hysteresis ratio h can be calculated by the ratio of the enclosed loop area of the curves to the enclosed area of loading curves, defined as:

$$h = \frac{\int_1^{\lambda_a} s_{loading} d\lambda - \int_1^{\lambda_a} s_{unloading} d\lambda}{\int_1^{\lambda_a} s_{loading} d\lambda} \quad \text{(S1)}$$

The stretch-dependent hysteresis ratio was measured by analyzing the stress-stretch curves obtained from cyclic loading and unloading tests conducted at progressively increasing stretches.

Measurement of fracture toughness. We characterized the fracture toughness Γ using pure-shear tests with both unnotched and notched samples (*9*). In the notched sample, a crack was introduced perpendicular to the loading direction, with its length measuring approximately one-fourth of the sample's width. Both samples were monotonically loaded to failure. The stress-stretch curves from unnotched samples can be used to evaluate the elastic energy density function $W(\lambda) = \int_1^{\lambda} s d\lambda$. The critical stretch λ_c was obtained from the notched sample, at which steady-state crack propagation occurs. The fracture toughness Γ was then calculated as $\Gamma = HW(\lambda_c) = H\int_1^{\lambda_c} s d\lambda$, where H is the initial height of the notched sample.

Measurement of fatigue threshold. We adopted the pure-shear tensile method to measure the fatigue thresholds of ELSPs at both high and low temperatures. Both unnotched and notched samples were employed in fatigue tests. First, the unnotched samples were cyclically loaded for 1000 cycles to measure the steady-state stretch-stress curves at different maximum applied stretches λ_a. The strain energy density W of the unnotched sample under the Nth cycle of a maximum applied stretch of λ_a was calculated as $W(\lambda_a, N) = \int_1^{\lambda_a} s(N) d\lambda$, where s and λ correspond to the steady-state nominal stress and stretch, respectively. Next, notched samples with the same dimensions were prepared with a one-fourth width crack and then cyclically stretched to the same λ_a for 1000 cycles. During this cyclic loading process, the crack extension Δc was recorded using a camera, and the crack extension rate dc/dN can be calculated subsequently. The corresponding applied energy release rate was determined as $G(\lambda_a, N) = \mathrm{H}\int_1^{\lambda_a} s(N) d\lambda$, where H is the height of the notched sample. We then plotted the curves of dc/dN versus G as shown in **Fig 3F, I**. By linearly extrapolating the curve of dc/dN versus G to the intercept with the abscissa, the measured fatigue threshold Γ_0 can be approximately identified. Because the ELSPs were transparent when immersed in a 55 °C oil bath, we added around 0.1% by volume of green food color to the mixture during the preparation process for better visualization.

<u>Elastocaloric test</u>

Elastocaloric tests were performed on a mechanical stage designed and built for controlling fiber deformation during stretching. Specimens were secured using 3D-printed grips, which were held at both ends by jaw clamps. One clamp was fixed, while the other was slidable along a guiding rail. To ensure accurate measurement of adiabatic elastocaloric performance, the tests were conducted at a high strain rate of 400 mm/s to minimize convection effects. A temperature-regulation chamber equipped with circulating anti-freezing working fluid and a thermoelectric (TE) module was used to maintain the specimens at temperatures above the melting point. The in-situ

temperature response was monitored using an infrared (IR) camera (FLIR ETS320) with a 320 × 240 IR sensor at a frame rate of 9 fps.

As illustrated in **Fig. S12A**, the elastocaloric test began with mounting a 3 mm segment (short segment) and regulating its center-point temperature to 65 °C by setting the temperature of the circulating working fluid to 70 °C and adjusting the voltage and current applied to the TE module. The fiber was then strained to a length of 30 mm (long segment). The specimen was held in the stretched state for 180 seconds and then released. The surface temperature of both the short and long segments was recorded along a software-generated fiber centerline throughout the entire duration of the measurement.

It should be noted that due to the openings at both ends for the placement of clamps, the temperature inside the chamber was not uniformly distributed. Specifically, the temperature at the center of the chamber was higher than that at the ends. During our experiments, the temperature at the ends (corresponding to the initial position of the short specimen) was controlled at 65 °C, while the center temperature (corresponding to the center of the long specimen) reached approximately 85 °C (**Fig. S12A**). Consequently, during stretching the specimen moves from the cooler end region toward the warmer center region. As shown in **Fig. 4A, B** and **Fig. S12B**, the average surface temperature exhibited an initial sharp increase from 66.1 °C to 71.8 °C within less than one second, driven by the occurrence of SIC. Following this adiabatic temperature rise, a gradual temperature increase was observed over several seconds, caused by heat exchange with the warmer chamber center. During release (**Fig. S12C**), the specimen exhibited an instantaneous adiabatic cooling followed by slow re-equilibration to the ambient chamber temperature.

To confirm that this slow temperature drift originated from environmental heat transfer rather than elastocaloric effects, a control experiment was performed using undeformed PLA under identical chamber conditions (**Fig. S12D**). No instantaneous temperature change was detected during stretching or releasing; instead, the PLA sample showed only gradual temperature drift due to thermal equilibration with the chamber environment (**Fig. S12E, F**). This comparison confirms that the gradual temperature variation observed in ELSPs arises from heat exchange, whereas the instantaneous temperature changes result from strain-induced crystallization.

To minimize potential measurement bias, we extracted the adiabatic temperature change (ΔT) by averaging temperature differences over the entire gauge region. As shown in **Fig. S13**, each pixel on the short segment was mapped onto a corresponding region on the long segment based on its relative position along the gauge length, and the average temperature of each group of pixels was calculated for each frame. The number of pixel groups in the long segment was the same as the number of pixels in the short segment, and these will be referred to as "points" hereafter. The heating temperature difference was obtained by subtracting the temperatures at each point on the short segment before stretching from those on the long segment afterward. Similarly, the cooling temperature difference was obtained by subtracting the temperatures at each point on the short

segment after release from those on the long segment before release. The average heating and cooling temperature differences were determined by calculating the corresponding mean values.

Supplementary Note

Thermodynamic model

We develop a thermodynamic model to describe the defect-regulated SIC and the corresponding uniaxial stress-strain response. The model builds on the pioneering work by Flory on SIC in natural rubbers (*10*) and adapts the recent continuum theory of Rastak and Linder (*11*). In addition, we assume that the presence of dangling chain defects induces two competing effects: (i) suppression of strain-induced crystallization due to reduced density of elastically active polymer chains; and (ii) promotion of cooling-induced crystallization due to enhanced flexibility of individual polymer chains.

In the current model, we focus on temperatures above or slightly below the melting temperature, such that the elasticity of stiff crystalline domains is neglected. At a prescribed temperature *T*, the density of total Helmholtz free energy of the elastomer consists of the entropic elastic energy $\psi_{\text{chain}}(\lambda_1, \lambda_2, \lambda_3, \chi)$ of the amorphous rubbery network and the energy of melting $\psi_{\text{c}}(\chi)$ of the crystalline domain:

$$\psi_{\text{total}}(\lambda_1, \lambda_2, \lambda_3, \chi) = \psi_{\text{chain}}(\lambda_1, \lambda_2, \lambda_3, \chi) + \psi_{\text{c}}(\chi) \tag{S2}$$

where λ_1, λ_2, and λ_3 are the three principal stretches, and χ is the crystallinity.

We model the elastic energy ψ_{chain} by adapting the Arruda-Boyce eight-chain model (*12*)

$$\psi_{\text{chain}}(\lambda_1, \lambda_2, \lambda_3, \chi) = \frac{kT\bar{\mu}}{\nu}\left[(1-\chi)\left(\log\left(\frac{\beta}{\sinh\beta}\right) + \frac{\beta}{\tanh\beta} - 1\right)\right] \tag{S3}$$

where k is the Boltzmann constant, $\nu = b^3$ is the volume of a single Kuhn segment, b is the Kuhn length, and $\beta(\lambda_1, \lambda_2, \lambda_3, \chi)$ is the dimensionless force in a polymer chain under stretch.

To incorporate the role of dangling chains in the suppression of strain-induced crystallization due to the reduced density of elastically active polymer chains, we introduce a dimensionless pre-factor $\bar{\mu}$ (*13*), expressed as

$$\bar{\mu} = 1 - \frac{5}{3}(1-P)^3 P - 7(1-P)^2 P^2 - \frac{17}{3}(1-P)P^3 - P^4 \tag{S4}$$

where P is the probability of forming a dangling chain for one macromer arm, related to the reaction efficiency p through

$$P = pP^3 + 1 - p \tag{S5}$$

In Eq. (S3), the dimensionless force $\beta(\lambda_1, \lambda_2, \lambda_3, \chi)$ is further related to the macroscopic stretches λ_i through the stretch of an individual chain Λ (*11, 12*):

$$\Lambda = \frac{\sqrt{{\lambda_1}^2+{\lambda_2}^2+{\lambda_3}^2}}{\sqrt{3}} = \sqrt{n}(1-\chi)\left(\frac{1}{\tanh}-\frac{1}{\beta}\right)+\sqrt{n}\chi \tag{S6}$$

where n is the number of Kuhn segments in a polymer chain. The two terms on the right-hand side of Eq. (S6) represent stretches of the amorphous rubbery phase and the crystalline phase, respectively.

The energy of melting $\psi_\mathrm{c}(\chi)$ is expressed as (*11*)

$$\psi_\mathrm{c}(\chi) = U_\mathrm{c}\left(\frac{T}{T_\mathrm{m,P}}-1\right)\chi - \xi\left[\frac{\chi}{\chi_\mathrm{max}}+\log\left(1-\frac{\chi}{\chi_\mathrm{max}}\right)\right] \tag{S7}$$

where U_c is the latent heat for crystallization, $T_\mathrm{m,P}$ is the melting temperature of a polymer network with a prescribed degree of defect P, $\xi > 0$ is a fitting constant, and χ_max is the maximum crystallinity. With ξ and χ_max, the second part of Eq. (S7) hence serves as a phenomenological energy barrier that limits the maximum crystallinity in the semicrystalline polymer (*11*).

To further incorporate the role of dangling chains in the promotion of cooling-induced crystallization due to the enhanced flexibility of individual polymer chains, we introduce a Landau-like approach (*14, 15*) to express the defect-dependent melting temperature as

$$T_\mathrm{m,P} = T_\mathrm{m}(1+\alpha P+\beta P^2) \tag{S8}$$

where T_m is the melting temperature of the defect-free network. We set the pre-factor $\alpha > 0$, such that the effective melting temperature $T_\mathrm{m,P}$ increases with the degree of defect P.

Finally, we assume the elastomer is incompressible, and consider the uniaxial stretch state, $\lambda_1 = \lambda$, $\lambda_2 = 1/\sqrt{\lambda}$, and $\lambda_3 = 1/\sqrt{\lambda}$. In equilibrium, thermodynamics dictates that

$$\frac{\partial\psi_\mathrm{total}(\lambda,\chi)}{\partial\chi} = 0 \tag{S9}$$

$$\frac{\partial\psi_\mathrm{total}(\lambda,\chi)}{\partial\lambda} = s \tag{S10}$$

where s is the nominal stress.

At $T = 60$ °C and $\lambda = 11$, we calculate the equilibrium crystallinity χ by solving Eq. (S9). At $T = 40$ °C and $\lambda = 1$, instead of directly applying $\lambda = 1$, we assume that the sample is stress-free, $s = 0$. This avoids an otherwise artificially introduced compressive force in the polymer chain due to the affine and isotropic deformation mapping in the 8-chain model. The equilibrium crystallinity χ is calculated by solving both Eq. (S9) and Eq. (S10).

In the model, we set $\nu = 1 \times 10^{-2}$ m^3, $n = 400$, and $U_\mathrm{c} = 3 \times 10^7$ J/m^3, on the same order of magnitude as the energy of fusion of rubbers reported in the literature (*16*). We further set $\xi = 10^5$ J/m^3 and $\chi_\mathrm{max} = 0.6$, similar to values in ref. (*11*). From the experiment, we set the melting temperature of the defect-free network to be $T_\mathrm{m} = 45$ °C. Finally, by fitting to the experimental

data in **Fig. 2F** ($T = 60$ °C and $\lambda = 11$) and **Fig. 2C** ($T = 40$ °C and $\lambda = 1$), we obtain $\alpha = 0.01$ and $\beta = 0.1$.

Difference between topological defects and crosslinking density

Topological defects and crosslinking density are two distinct concepts in polymer networks. Topological defects refer to structural irregularities such as dangling chains and cyclic loops (*17, 18*). These defects influence material properties in unique ways that intrinsically differ from crosslinking density, which describes the molar ratio between crosslinks and monomers. Using shear modulus as one example, following Eqs. (S3)&(S4), the shear modulus μ of a polymer network is determined by (*13*)

$$\mu = \bar{\mu}NkT = NkT\left[1 - \frac{5}{3}(1-P)^3P - 7(1-P)^2P^2 - \frac{17}{3}(1-P)P^3 - P^4\right] \quad \text{(S11)}$$

where $N = 1/(nv)$ is the number of elastically effective chains per unit volume. Therefore, the crosslinking density, represented by N, is governed by the polymer chain length n through individual macromers. By contrast, the topological defect density, represented by P, is governed by the order and density of dangling-chain defects through the reaction efficiency p and hydrolysis time (*1, 13, 19*). In this work, topological defects and crosslinking density synergistically modulate the polymer network's entropic elasticity, thereby impacting its elastocaloric effect.

Table S1 summarizes how variations in crosslinking density and topological defects influence mechanical properties and elastocaloric performance. While increasing chemical crosslinking density may improve the overall homogeneity of network structure, it also makes the material less stretchable, suppressing nonlinear elastic deformation. This reduction in stretchability can hinder the elastocaloric potential, as elastocaloric performance also relies on the ability of the material to undergo large deformation, which is crucial for maximizing adiabatic temperature changes. Rather than a simple correlation between crosslinking density and elastocaloric performance, topological defects introduce a more complex impact by simultaneously suppressing SIC and promoting TIC, leading to a non-monotonic elastocaloric response.

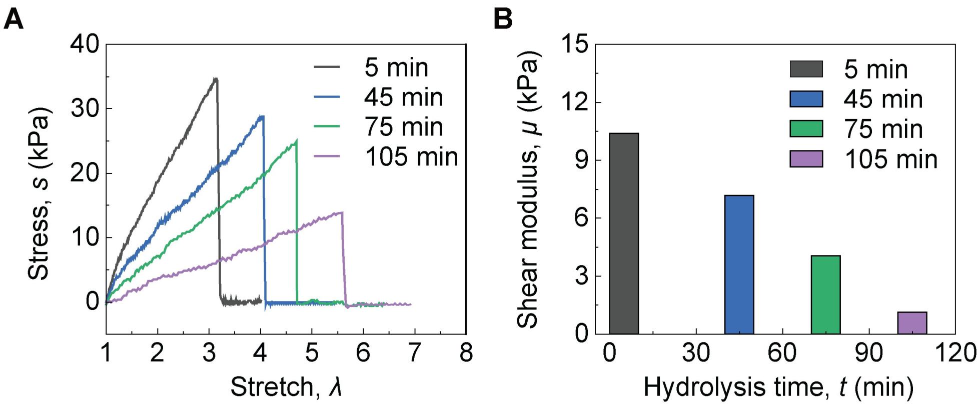

Figure S1 Mechanical properties of end-linked star hydrogel with various hydrolysis times. (A) Nominal stress-stretch curves and **(B)** corresponding shear modulus μ of end-linked star hydrogel with various hydrolysis times. The modulus and strength decrease as hydrolysis time increases from 5 min to 105 min due to the increase in density of defects.

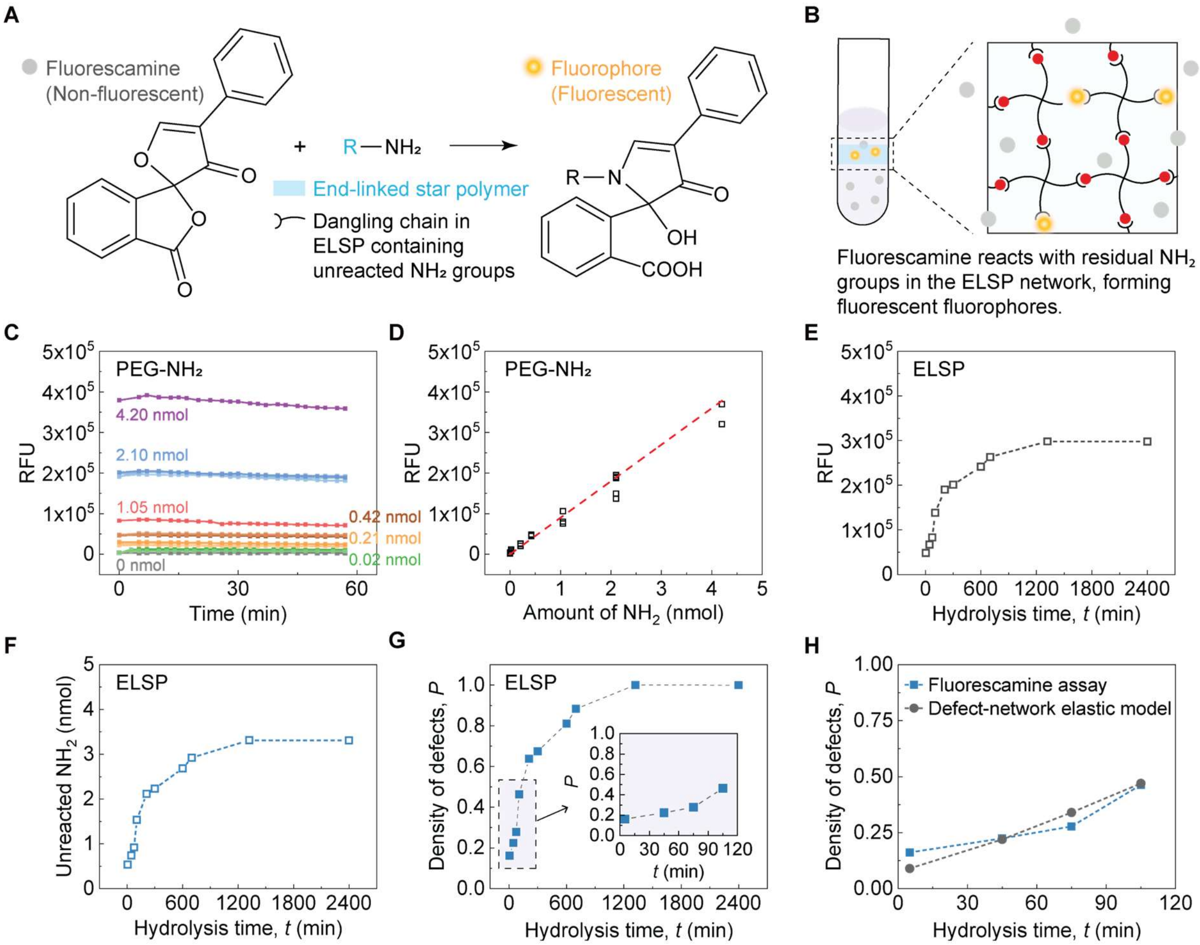

Figure S2 Quantitative determination of residual unreacted amine groups and density of defects in ELSP using the fluorescamine assay. (A) Reaction between fluorescamine and amine groups forms fluorescent fluorophores. **(B)** Schematic illustration of the working mechanism of fluorescamine assay, where fluorescamine reacts with residual NH_2 groups in the ELSP network to form localized fluorescent sites. **(C)** Relative fluorescence units (RFU) measured for PEG-NH_2 (20 kDa) solutions with known amine contents. **(D)** Calibration curve obtained from (C), showing a strong linear correlation between RFU and NH_2 amount below 4 nmol (red line). **(E)** Measured RFU values of ELSP samples prepared with different hydrolysis times. **(F)** Corresponding unreacted NH_2 contents in ELSP with different hydrolysis time determined from the calibration curve in (D). Longer hydrolysis times lead to more extensive NHS hydrolysis, resulting in a higher amount of unreacted amines and eventually reaching a plateau. **(G)** Density of defects in the ELSP network as a function of hydrolysis time, calculated by normalizing the residual NH_2 content in (F) to the plateau value. **(H)** Comparison between the density of defects determined by the fluorescamine assay and the defect-network elastic model, showing good agreement.

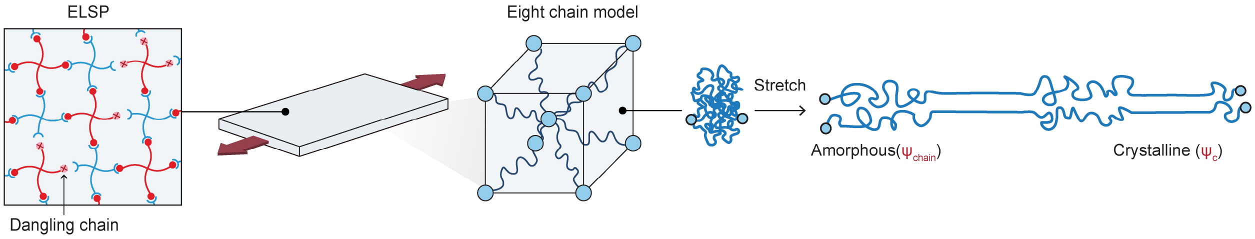

Figure S3 Theoretical framework of the thermodynamic model. The model describes the defect-regulated SIC and the corresponding uniaxial stress-strain responses by integrating three theoretical frameworks: 1) eight-chain model, 2) ideal chain model, and 3) crystallization model.

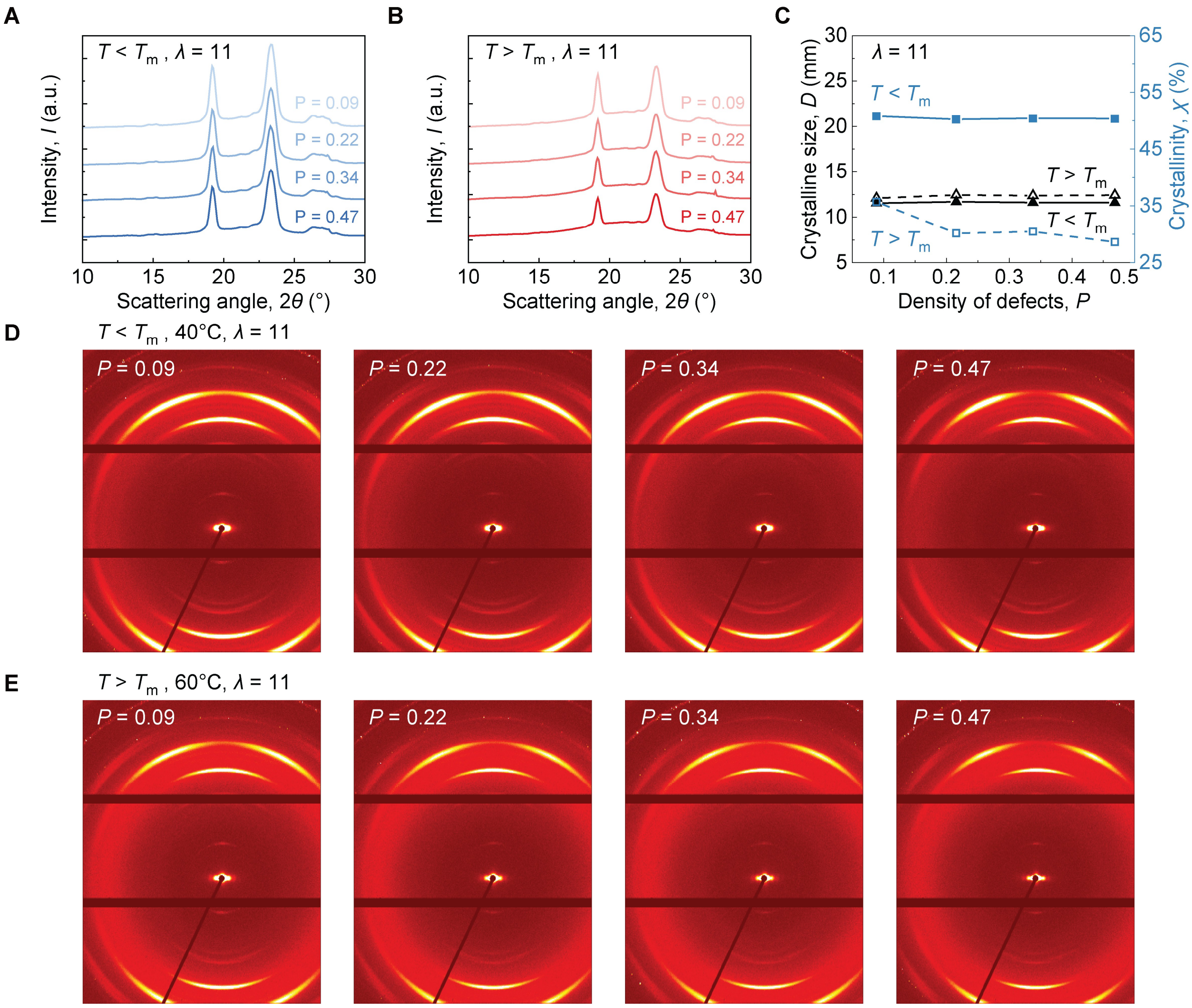

Figure S4 WAXS scans of highly stretched ELSPs with various densities of dangling-chain defects. (A) 1D radial WAXS profiles of highly stretched ELSPs ($\lambda = 11$) with various densities of defects below T_m at 40 °C averaged over all azimuthal angles. **(B)** 1D radial WAXS profiles of highly stretched ELSPs ($\lambda = 11$) with various densities of defects above T_m at 60 °C averaged over all azimuthal angles. **(C)** As density of defects increases, the crystalline size remains of ELSPs stable, while the crystallinity stays constant below T_m but decreases above T_m. **(D)** WAXS scattering patterns of ELSPs with various densities of defects at temperatures below T_m at 40 °C. **(E)** WAXS scattering patterns of ELSPs with various densities of defects at temperatures above T_m at 60 °C.

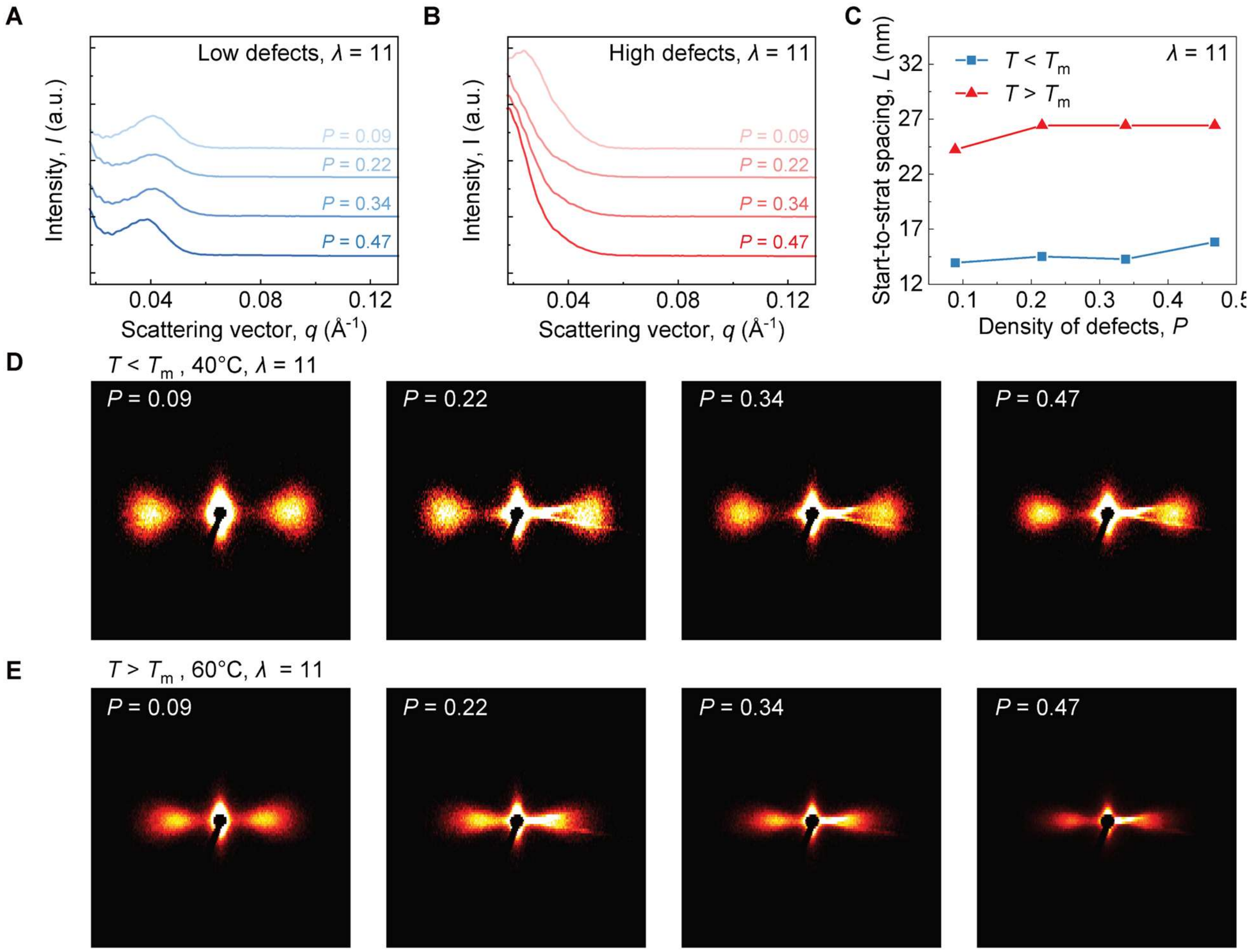

Figure S5 SAXS scans of highly stretched ELSPs with various densities of dangling-chain defects. **(A)** 1D radial SAXS profiles of highly stretched ELSPs ($\lambda = 11$) with various densities of dangling-chain defects below T_m at 40 °C. **(B)** 1D radial SAXS profiles of highly stretched ELSPs ($\lambda = 11$) with various densities of dangling-chain defects above T_m at 60 °C. **(C)** The start-to-start spacing L between adjacent crystalline domains for ELSPs with various densities of dangling-chain defects. **(D)** SAXS scattering patterns of ELSPs with various densities of dangling-chain defects at temperatures below T_m at 40 °C. **(E)** SAXS scattering patterns of ELSPs with various densities of dangling-chain defects at temperatures above T_m at 60 °C.

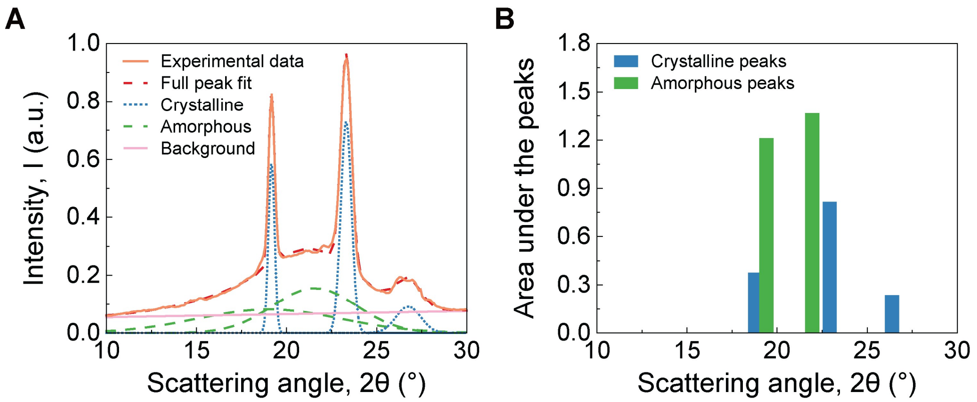

Figure S6 1D WAXS curve peak fitting scheme. **(A)** Crystallinity is determined by averaging 2D WAXS scans across all azimuthal angles to produce a single 1D curve. **(B)** Crystallinity is calculated from the ratio of the total area under the crystalline peaks to the combined area of crystalline and amorphous peaks. This figure shows the WAXS curve peak fitting and crystallinity calculation of highly stretched ELSPs with low defects (P = 0.09, λ = 11) above T_m at 60 °C.

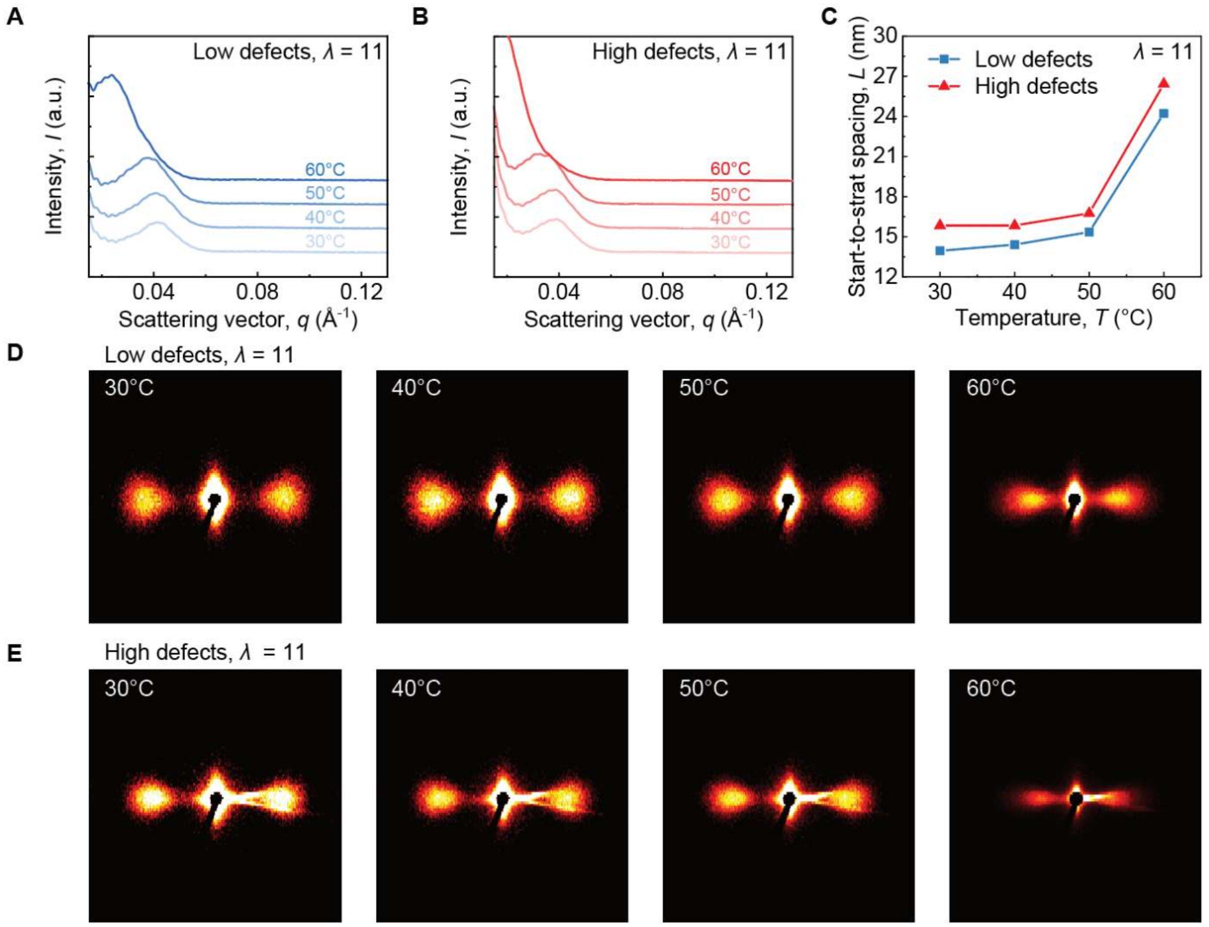

Figure S7 SAXS scans of highly stretched ELSPs at different temperatures. (A) 1D radial SAXS profiles of highly stretched ELSPs (λ = 11) with low defects (P = 0.09) at various temperatures from 30 °C to 60 °C. **(B)** 1D radial SAXS profiles of highly stretched ELSPs (λ = 11) with high defects (P = 0.47) at various temperatures from 30 °C to 60 °C. **(C)** The start-to-start spacing L between adjacent crystalline domains for ELSPs as temperature increases from below T_{m} to above T_{m}. **(D)** SAXS scattering patterns of ELSPs at various temperatures for samples with low defects (P = 0.09). **(E)** SAXS scattering patterns of ELSPs at various temperatures for samples with high defects (P = 0.47).

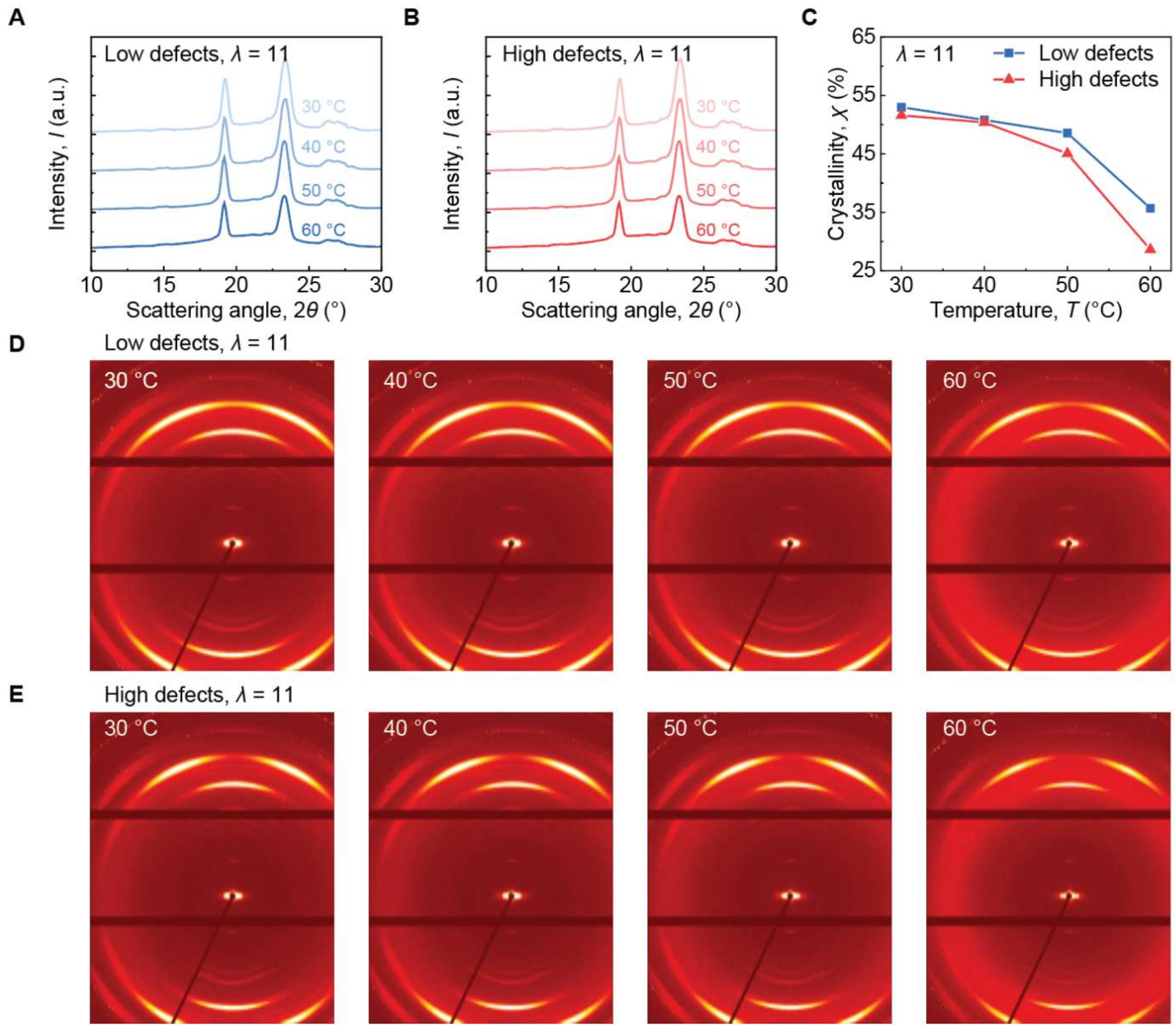

Figure S8 WAXS scans of highly stretched ELSPs at different temperatures. (A) 1D radial WAXS profiles of highly stretched ELSPs (λ = 11) with low defects (P = 0.09) at various temperatures from 30 °C to 60 °C, averaged over all azimuthal angles. **(B)** 1D radial WAXS profiles of highly stretched ELSPs (λ = 11) with high defects (P = 0.47) at various temperatures from 30 °C to 60 °C, averaged over all azimuthal angles. **(C)** Crystallinity of ELSPs significantly decreases as temperature increases from below T_m to above T_m. **(D)** WAXS scattering patterns of ELSPs at various temperatures for samples with low defects (P = 0.09). **(E)** WAXS scattering patterns of ELSPs at various temperatures for samples with high defects (P = 0.47).

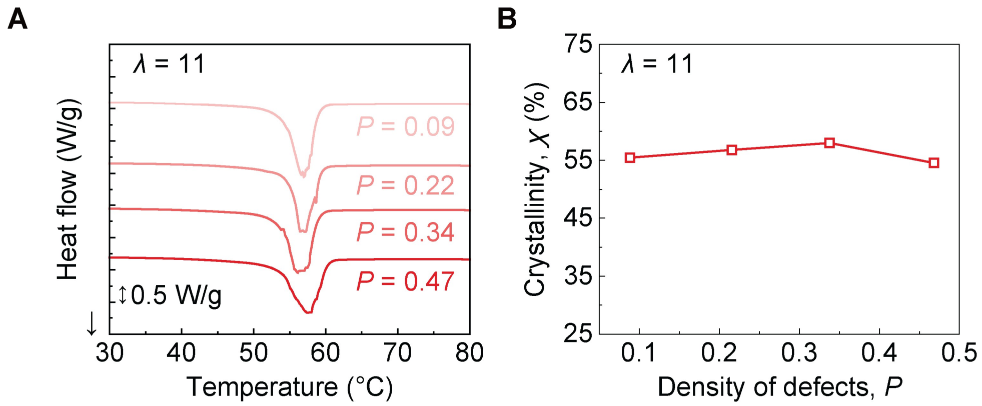

Figure S9 Differential scanning calorimetry (DSC) characterization of ELSPs with various densities of dangling-chain defects. (A) DSC curves of ELSPs at highly stretched state ($\lambda = 11$). **(B)** Crystallinity of highly stretched ELSPs remains nearly stable as density of defects increases.

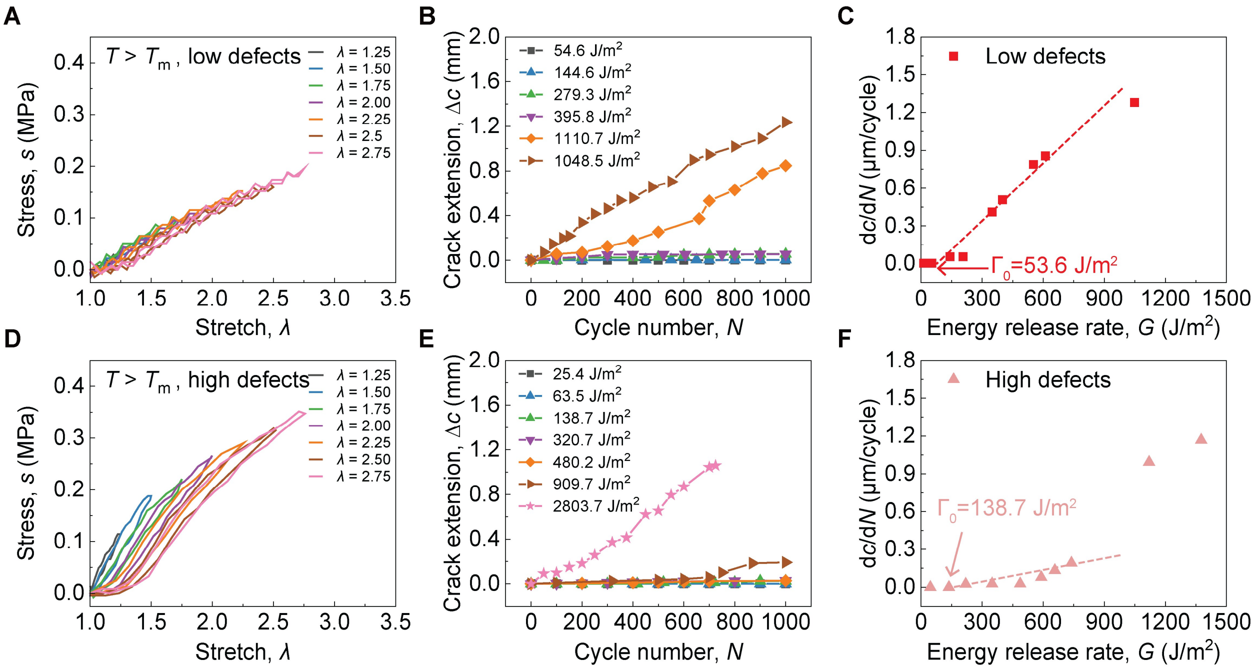

Figure S10 Measurement of fatigue threshold of ELSPs above T_m. (A) Steady-state nominal stress versus stretch curves of ELSPs with low defects (P = 0.09) under the applied stretch from 1.25 to 2.75. **(B)** Crack extension Δc versus cycle number N of ELSPs with low defects (P = 0.09) under the applied stretch from 1.25 to 2.75. **(C)** Crack extension per cycle dc/dN versus applied energy release rate G of ELSPs with low defects (P = 0.09). The fatigue threshold of ELSPs with low defects (P = 0.09) above T_m is 53.6 J/m^2. **(D)** Steady-state nominal stress versus stretch curves of ELSPs with high defects (P = 0.47) under the applied stretch from 1.25 to 2.75. **(E)** Crack extension Δc versus cycle number N of ELSPs with high defects (P = 0.47) under the applied stretch from 1.25 to 2.75. **(F)** Crack extension per cycle dc/dN versus applied energy release rate G of ELSPs with high defects (P = 0.47). The fatigue threshold of ELSPs with high defects (P = 0.47) above T_m is 138.7 J/m^2.

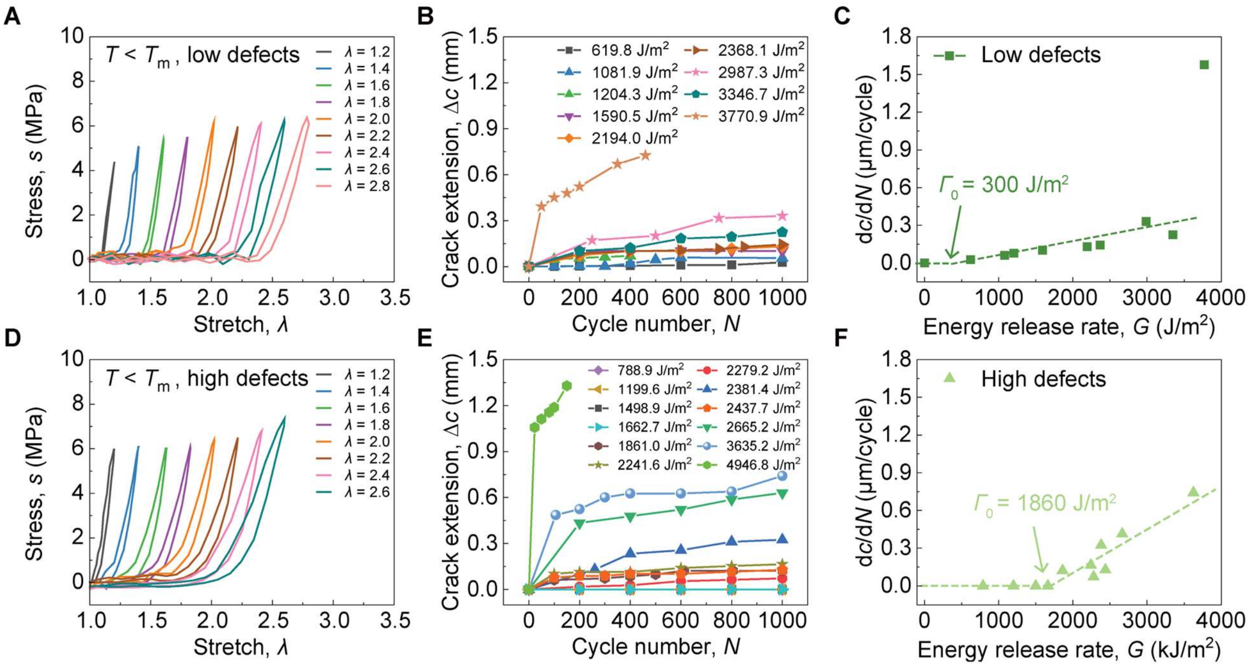

Figure S11 Measurement of fatigue threshold of ELSPs below T_m. (A) Steady-state nominal stress versus stretch curves and **(B)** Crack extension Δc versus cycle number N of ELSPs with low defects (P = 0.09) under the applied stretch from 1.2 to 2.8. **(C)** Crack extension per cycle dc/dN versus applied energy release rate G of ELSPs with low defects (P = 0.09). The fatigue threshold of ELSPs with low defects (P = 0.09) below T_m is 300 J/m^2. **(D)** Steady-state nominal stress versus stretch curves and **(E)** Crack extension Δc versus cycle number N of ELSPs with high defects (P = 0.47) under the applied stretch from 1.2 to 2.6. **(F)** Crack extension per cycle dc/dN versus applied energy release rate G of ELSPs with high defects (P = 0.47). The fatigue threshold of ELSPs with high defects (P = 0.47) below T_m is 1861 J/m^2.

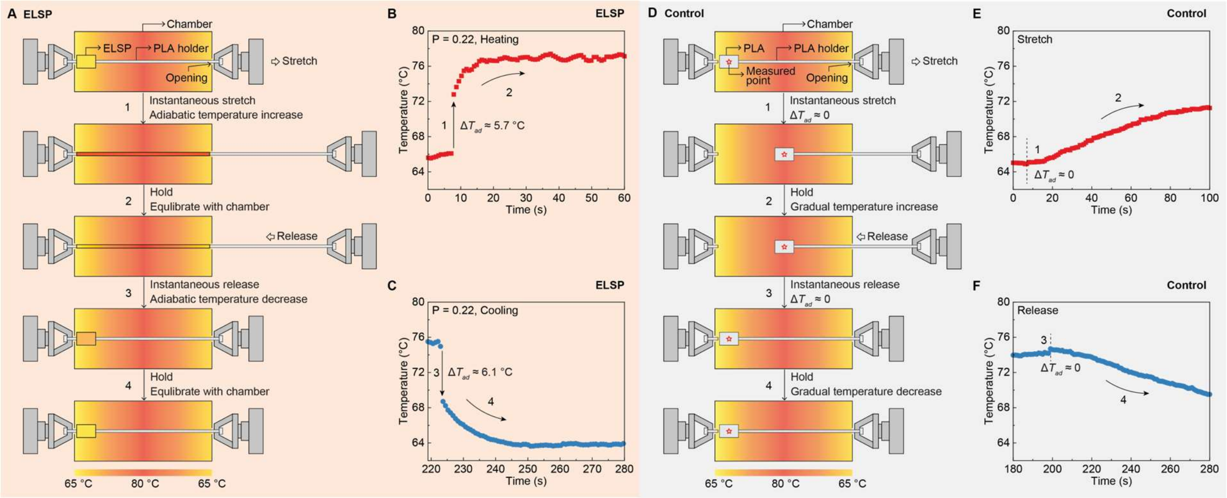

Figure S12 Schematic of elastocaloric experiments and corresponding temperature changes during stretching and releasing processes for ELSP and control verification. (A) Schematic illustration of the elastocaloric experiment for ELSPs under stretching and releasing within a heated chamber. The chamber has openings at both ends to accommodate clamps, resulting in a temperature gradient (~65 °C near the ends and ~85 °C at the center). **(B)** Measured surface temperature of the ELSP with moderate defect density (P = 0.22) during instantaneous stretching and subsequent equilibration. **(C)** Measured surface temperature of the ELSP with moderate defect density (P = 0.22) during instantaneous releasing and subsequent equilibration. **(D)** Schematic of the control experiment using undeformed PLA under identical chamber conditions to isolate the effect of heat exchange with the chamber environment. **(E)** Measured surface temperature of the PLA during instantaneous stretching and subsequent equilibration. **(F)** Measured surface temperature of the PLA during instantaneous releasing and subsequent equilibration.

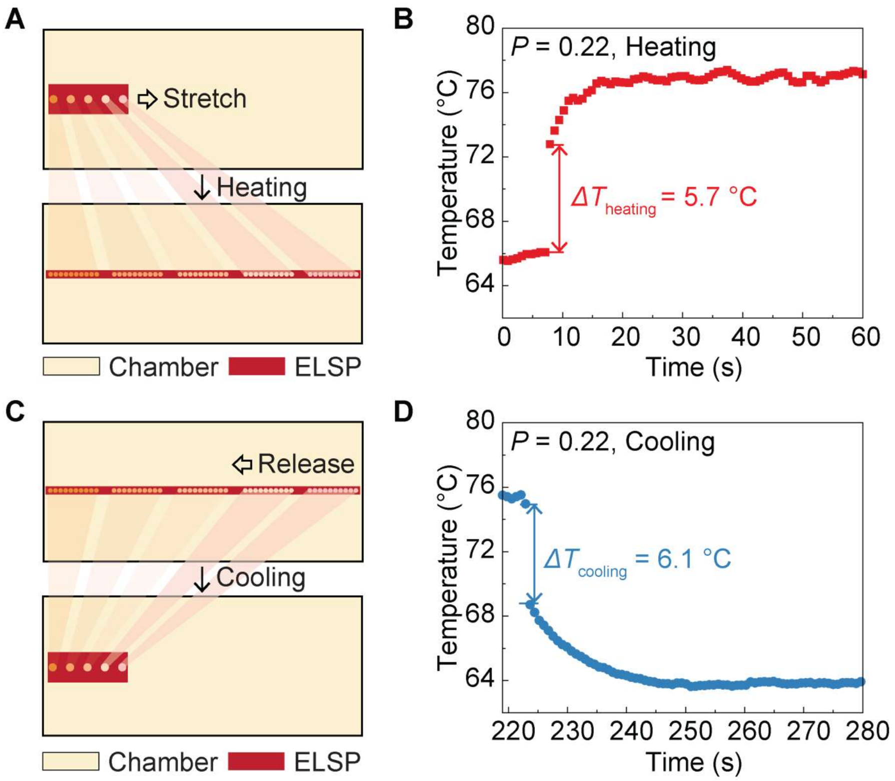

Figure S13 Schematic of elastocaloric experiments and corresponding temperature changes during stretching and releasing processes. (A) Schematic illustration of the stretching process. **(B)** Measured surface temperature of the ELSP with moderate defect density (P = 0.22) during instantaneous stretching. **(C)** Schematic illustration of the releasing process. **(D)** Measured surface temperature of the ELSP with moderate defect density (P = 0.22) during instantaneous releasing.

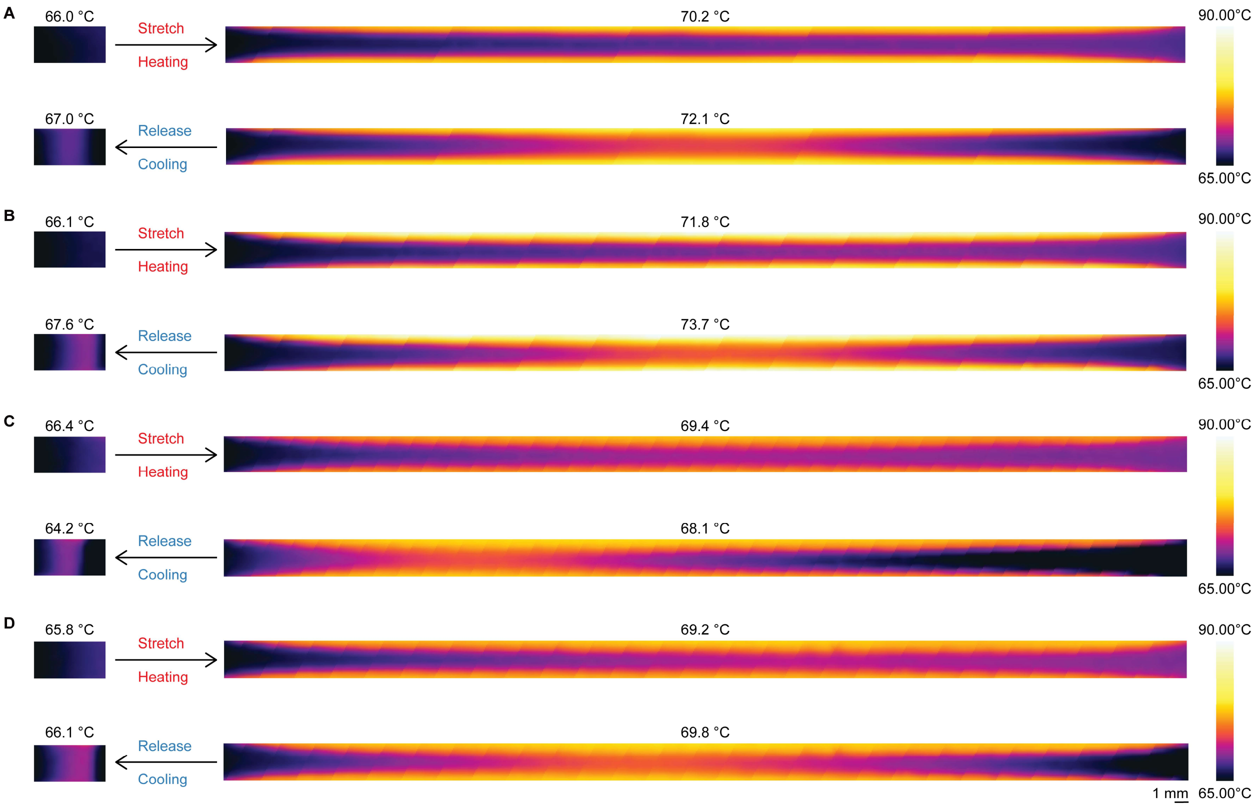

Figure S14 Elastocaloric experiments for ELSPs with various densities of dangling-chain defects. Thermal images of ELSPs with various densities of defects during adiabatic stretching and releasing processes: **(A)** $P = 0.09$, **(B)** $P = 0.22$, **(C)** $P = 0.34$, and **(D)** $P = 0.47$. All samples exhibit a temperature increase during stretching and a decrease during releasing. As density of defects increases, the temperature change first increases and then decreases, with the peak temperature change observed at $P = 0.22$.

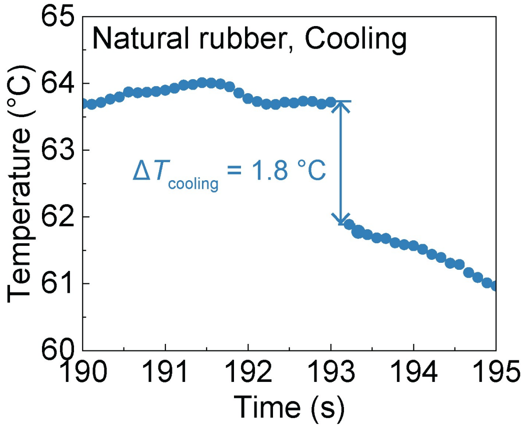

Figure S15 Elastocaloric performance of natural tubber. Measured surface temperature of natural rubber during instantaneous releasing.

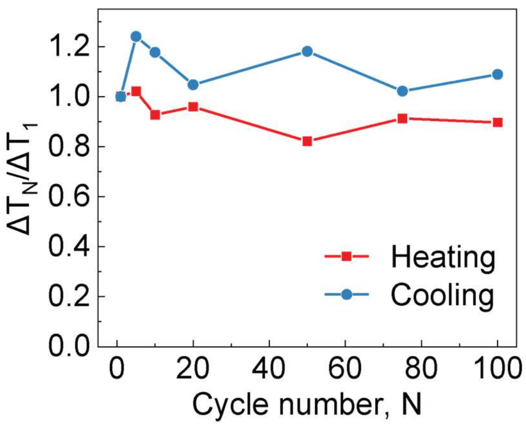

Figure S16 Normalized adiabatic temperature change ($\Delta T_N/\Delta T_1$) of ELSPs with low defects across 100 cycles. The elastocaloric performance remains relatively stable.

Table S1 Influence of topological defect density and crosslinking density on mechanical property and elastocaloric performance.

Topological defects density	Crosslinking density	Modulus	Stretchability	Strength	Elastocaloric performance
Low	Low	Low	High	High	High (*2*)
Low	High	High	Low	Moderate	Low
High	Low	Low	High	Moderate	Moderate*
High	High	Low	Low	Low	Low

* Our data show that there is a non-monotonic relationship between defect density and elastocaloric performance. Incorporating low-density dangling-chain defects leads to a slight improvement in the elastocaloric effect, while high-density dangling-chain defects result in a slight reduction.

Table S2. Fundamental studies on elastocaloric materials

**Reference**	**Material system**	**Primary focus**	**Randomly crosslinked polymer network**	**End-linked polymer network**
Advanced Science, 11, 12, 2307741, 2024	Natural rubber	The interplay between local strain field and SIC	✓	×
Applied Thermal Engineering, 57, 33-38, 2013	Natural rubber	Modeling the strain-temperature relationship for elastocaloric effect	✓	×
Macromolecules, 53, 7, 2606-2615, 2020	Natural rubber	The origin of giant barocaloric effect	✓	×
Liquid Crystals, 48, 3, 405-411, 2021	Liquid crystal elastomer	Impact of crosslinking density	✓	×
Journal of Physics: Energy, 5, 4, 2023	Liquid crystal elastomer	Impact of crosslinking density	✓	×
Journal of Molecular Liquids, 413, 126058, 2024	Liquid crystal elastomer	Theoretical modeling of elastocaloric responses in nematic elastomers under adiabatic uniaxial stress	✓	×
Nat Commun, 15, 6567, 2024	Graphene nanoplatelets / polystyrene-*b*-poly(ethylene-*co*-butylene)-*b*-polystyrene (SEBS)	Enhancement from shearo-caloric effect	✓	×
Nat Commun, 13, 9, 2022	Commercial triblock poly(styrene-b-ethylene-co-butylene-b-styrene) (SEBS) / polystyrene (PS) blocks	Impact of chain length	✓	×
Our work	End-linked star-shaped PEG elastomer (ELSP)	Impact of topological defects	×	✓

Table S3 Comparison of the relationship between elastocaloric performance and ambient temperature in various polymers under tensile testing

Material system	Ambient temperature	Strain or stress	Adiabatic temperature change	Reference
Commercial triblock poly(styrene-b-ethylene-co-butylene-b-styrene) (SEBS)	25 °C	6	15.3 °C	*Nat Commun*, 13, 9, 2022
Thermoplastic polyurethane (TPU)	25 °C	4	7.7 °C	*Appl. Phys. Lett.,* 117, 193903, 2020
Nontwisted polyethylene (PE)	25 °C	0.227	< 0.1 °C	*Science*, 366, 6462, 216-221, 2019
P(VDF-TrFE-CTFE)	27 °C	0.12	2.15 °C	*Appl. Phys. Lett.*, 108, 242904, 2016
PVDF-based polymers	25 °C	10 MPa	1.8 °C	*Appl. Phys. Lett.*, 108, 072903, 2016
Main chain liquid crystal elastomer (LCE)	67 °C	0.75	0.35 °C	*Philos Trans A Math Phys Eng Sci*, 374, 2074, 20150301, 2016
Main chain liquid crystal elastomer (LCE)	~ 125 °C	0.9	~ 1 °C	*Liquid Crystals*, 48, 3, 405-411, 2021
Main chain liquid crystal elastomer (LCE)	64 °C	0.38	0.11 °C	*Phil. Trans. R. Soc. A.*, 374, 20150301
	67 °C	0.38	0.18 °C	
	70 °C	0.38	0.1 °C	
	67 °C	0.75	0.35 °C	
Main chain liquid crystal elastomer (LCE)	23 °C	1.5	3.42 °C	*Small*, 20, 32, 2400786, 2024
Main-chain liquid crystal monomer C6M / BPADA	25 °C	1	2.3 °C	*Phys. Rev. Materials*, 5, L062401, 2021
Latex rubber	24 °C	7	14 °C	*Tech. Phys.*, 61, 1679-1683, 2016
Nitrile butadiene rubber	50 °C	26 MPa	1.3 °C	*ACS Applied Polymer Materials*, 1, 8, 2019
Natural rubber / sulphur	25 °C	6.2	6 °C	*Macromol. Rapid Commun.*, 45, 2400422, 2024
Natural rubber / dicumyl peroxide	25 °C	6.8	10.8 °C	
Natural rubber / ground tyre rubber blends	25 °C	6.3	11.4 °C	
Natural rubber with pre-elongation of 4	25 °C	0.69	4.3 °C	*Appl. Phys. Lett.*, 107, 081905, 2015
Natural rubber	25 °C	6	8.8 °C	*Appl. Phys. Lett.*, 108, 041901, 2016
Natural rubber	20 °C	5	4.6 °C	*J. Phys. Energy*, 6, 025003, 2024
Natural rubber balloon	25 °C	~ 6.6 kPa	~ 7.9 °C	*Nature Energy*, 6, 260-267, 2021
Natural rubber fibers	25 °C	3	2.4 °C	*Science*, 366, 6462, 216-221, 2019
	25 °C	6	12.2 °C	
Natural rubber	49 °C	6	6 °C	*Physics Letters A*, 381, 25-26, 2112-2116, 2017
Natural rubber	55 °C	10	3.5 °C	*Science Advances*, 9, 50, 2023
ELSP	55 °C	10	9.3 °C	
Natural rubber	65 °C	10	1.8 °C	*This work*
ELSP	70 °C	10	6 °C	*This work*

Reference

1. S. Lin, J. Ni, D. Zheng, X. Zhao, Fracture and fatigue of ideal polymer networks. *Extreme Mechanics Letters* **48**, 101399 (2021).
2. C. M. Hartquist *et al.*, An elastomer with ultrahigh strain-induced crystallization. *Science Advances* **9**, eadj0411 (2023).
3. C. M. Hartquist *et al.*, Reversible two-way tuning of thermal conductivity in an end-linked star-shaped thermoset. *Nature Communications* **15**, 5590 (2024).
4. T. Matsunaga, T. Sakai, Y. Akagi, U.-i. Chung, M. Shibayama, Structure Characterization of Tetra-PEG Gel by Small-Angle Neutron Scattering. *Macromolecules* **42**, 1344-1351 (2009).
5. S. M. Derayea, E. Samir, A review on the use of fluorescamine as versatile and convenient analytical probe. *Microchemical Journal* **156**, 104835 (2020).
6. S. Udenfriend *et al.*, Fluorescamine: A Reagent for Assay of Amino Acids, Peptides, Proteins, and Primary Amines in the Picomole Range. *Science* **178**, 871-872 (1972).
7. Y. Golitsyn *et al.*, Crystallization in PEG networks: The importance of network topology and chain tilt in crystals. *Polymer* **165**, 72-82 (2019).
8. K. Frost, D. Kaminski, G. Kirwan, E. Lascaris, R. Shanks, Crystallinity and structure of starch using wide angle X-ray scattering. *Carbohydrate Polymers* **78**, 543-548 (2009).
9. R. S. Rivlin, A. G. Thomas, Rupture of rubber. I. Characteristic energy for tearing. *Journal of Polymer Science* **10**, 291-318 (1953).
10. P. J. Flory, Thermodynamics of Crystallization in High Polymers. I. Crystallization Induced by Stretching. *The Journal of Chemical Physics* **15**, 397-408 (1947).
11. R. Rastak, C. Linder, A non-affine micro-macro approach to strain-crystallizing rubber-like materials. *Journal of the Mechanics and Physics of Solids* **111**, 67-99 (2018).
12. E. M. Arruda, M. C. Boyce, A three-dimensional constitutive model for the large stretch behavior of rubber elastic materials. *Journal of the Mechanics and Physics of Solids* **41**, 389-412 (1993).
13. S. Lin, X. Zhao, Fracture of polymer networks with diverse topological defects. *Physical Review E* **102**, 052503 (2020).
14. N. Provatas, K. Elder, *Phase-field methods in materials science and engineering*. (John Wiley & Sons, 2011).
15. R. Bai, E. Ocegueda, K. Bhattacharya, Photochemical-induced phase transitions in photoactive semicrystalline polymers. *Physical Review E* **103**, 033003 (2021).
16. J. Plagge, M. Klüppel, Determining strain-induced crystallization of natural rubber composites by combined thermography and stress-strain measurements. *Polymer Testing* **66**, 87-93 (2018).
17. M. Zhong, R. Wang, K. Kawamoto, B. D. Olsen, J. A. Johnson, Quantifying the impact of molecular defects on polymer network elasticity. *Science* **353**, 1264-1268 (2016).
18. H. Zhou *et al.*, Counting primary loops in polymer gels. *Proceedings of the National Academy of Sciences* **109**, 19119-19124 (2012).
19. Y. Akagi, T. Matsunaga, M. Shibayama, U.-i. Chung, T. Sakai, Evaluation of Topological Defects in Tetra-PEG Gels. *Macromolecules* **43**, 488-493 (2010).
